# Authentication of Polish Red Wines Produced from Zweigelt and Rondo Grape Varieties Based on Volatile Compounds Analysis in Combination with Machine Learning Algorithms: Hotrienol as a Marker of the Zweigelt Variety

**DOI:** 10.3390/molecules28041961

**Published:** 2023-02-18

**Authors:** Anna Stój, Tomasz Czernecki, Dorota Domagała

**Affiliations:** 1 Department of Biotechnology, Microbiology and Human Nutrition, Faculty of Food Science and Biotechnology, University of Life Sciences, 8 Skromna Street, 20-704 Lublin, Poland; 2 Department of Applied Mathematics and Computer Science, Faculty of Production Engineering, University of Life Sciences in Lublin, 28 Głęboka Street, 20-612 Lublin, Poland

**Keywords:** wine adulteration, 3,7-dimethyl-1,5,7-octatrien-3-ol, HS-SPME/GC-MS, support vector machine, k-nearest neighbor

## Abstract

The aim of this study was to determine volatile compounds in red wines of Zweigelt and Rondo varieties using HS-SPME/GC-MS and to find a marker and/or a classification model for the assessment of varietal authenticity. The wines were produced by using five commercial yeast strains and two types of malolactic fermentation. Sixty-seven volatile compounds were tentatively identified in the test wines; they represented several classes: 9 acids, 24 alcohols, 2 aldehydes, 19 esters, 2 furan compounds, 2 ketones, 1 sulfur compound and 8 terpenes. 3,7-dimethyl-1,5,7-octatrien-3-ol (hotrienol) was found to be a variety marker for Zweigelt wines, since it was detected in all the Zweigelt wines, but was not present in the Rondo wines at all. The relative concentrations of volatiles were used as an input data set, divided into two subsets (training and testing), to the support vector machine (SVM) and k-nearest neighbor (kNN) algorithms. Both machine learning methods yielded models with the highest possible classification accuracy (100%) when the relative concentrations of all the test compounds or alcohols alone were used as input data. An evaluation of the importance value of subsets consisting of six volatile compounds with the highest potential to distinguish between the Zweigelt and Rondo varieties revealed that SVM and kNN yielded the best classification models (F-score of 1, accuracy of 100%) when 3-ethyl-4-methylpentan-1-ol or 3,7-dimethyl-1,5,7-octatrien-3-ol (hotrienol) or subsets containing one or both of them were used. Moreover, the best SVM model (F-score of 1) was built with a subset containing 2-phenylethyl acetate and 3-(methylsulfanyl)propan-1-ol.

## 1. Introduction

The wine sector is one of the most profitable agri-food industries [1,2,3]. The price of wine should reflect its quality, which is influenced by the grape variety, terroir, vintage, age and the style of wine. The wide price range creates opportunities for fraud aimed at achieving greater profit. The methods of detecting wine adulteration rely on determining deviations from the standard contents of natural components or demonstrating the presence of a foreign component (a marker). Both methods are commonly used in scientific research, but the latter is more accurate. Wine authentication is based on identity verification to ensure that the product is as declared on the label [1].

Varietal adulteration of wine is defined as the addition of must produced from grape varieties other than the labelled variety in illegal quantities [4]. It can be detected by volatile compound analysis. The volatile compounds determining the aroma of wine originate from grapes (varietal aromas) and are secondary products of fermentation processes (fermentation aromas) and aging (post-fermentation aromas) [5]. The compounds derived from grapes provide varietal differentiation. Some volatile compounds synthesized in grapes exist in volatile forms but mostly are non-volatile aroma precursors, which are released through biochemical and chemical reactions during fermentation and aging [3,6,7]. These compounds include monoterpenes, C13-norisoprenoids, C6-compounds, methoxypyrazines and mercaptans [6,8,9]. Forty important monoterpenes are found in grapes, including the following monoterpene alcohols and oxides: geraniol, linalool, citronellol, nerol, (*E*)-hotrienol and cis- or (*Z*)-rose oxide, which have floral aromas [6].

There are two approaches to the analysis of volatile compounds: targeted analysis and non-targeted analysis. Targeted analysis concerns selected compounds that are relevant to the given research problem, while non-targeted analysis aims to determine as many compounds as possible and creates patterns, which can be used to differentiate samples using statistical methods. The non-targeted approach is more appropriate for the study of potential markers of wine authenticity [10].

Non-targeted analysis of volatile compounds in combination with conventional statistical methods, such as principal component analysis (PCA), hierarchical component analysis (HCA) and linear discriminant analysis (LDA), has been employed for varietal differentiation of red wines [8,11,12,13,14]. Alternative data mining based on machine learning (ML) algorithms has a high potential for varietal authentication [15]. SVM [16] and directed acyclic graph (DAG) decision tree [17] have been applied to explore the volatiles’ fingerprints of red wines. In addition, the following machine learning methods have been used to analyze volatile compounds in white wines: SVM, random forest (RF), multilayer perceptron (MLP), kNN and naive Bayes (NB) [18]. Other examples of varietal authentication strategies include phenolic compounds/RF [19], total phenolic, flavonoid, anthocyanin and tannin content/artificial neural networks (ANN) [20], NMR spectra/RF [21], NIR spectra/radial basis function neural networks (RBFNN) and least-squares support vector machines (LS-SVM) [22], as well as fluorescence spectra/extreme gradient boosting discriminant analysis (XGBDA) [23].

The OIV regulates the rules for indicating the name of the grape variety (or varieties) on the wine label, but this information is optional [24]. The law lists the names of the varieties authorized for the production, labeling and presentation of wine [25] but does not provide the values of parameters and/or markers that could be used for the varietal authentication of wines. In Poland, there is an opportunity for making false declarations of grape variety, i.e., for designating a wine from the Rondo variety, which is one of the most commonly grown varieties of red grapes, as a wine from the Zweigelt variety, which is less commonly grown [26]. The aim of the present study was to determine volatile compounds in red wines of the Zweigelt and Rondo varieties using HS-SPME/GC-MS and to find a marker and/or a classification model for the assessment of their varietal authenticity regardless of yeast strain and type of malolactic fermentation (MLF).

## 2. Results and Discussion

**Figure 1:**
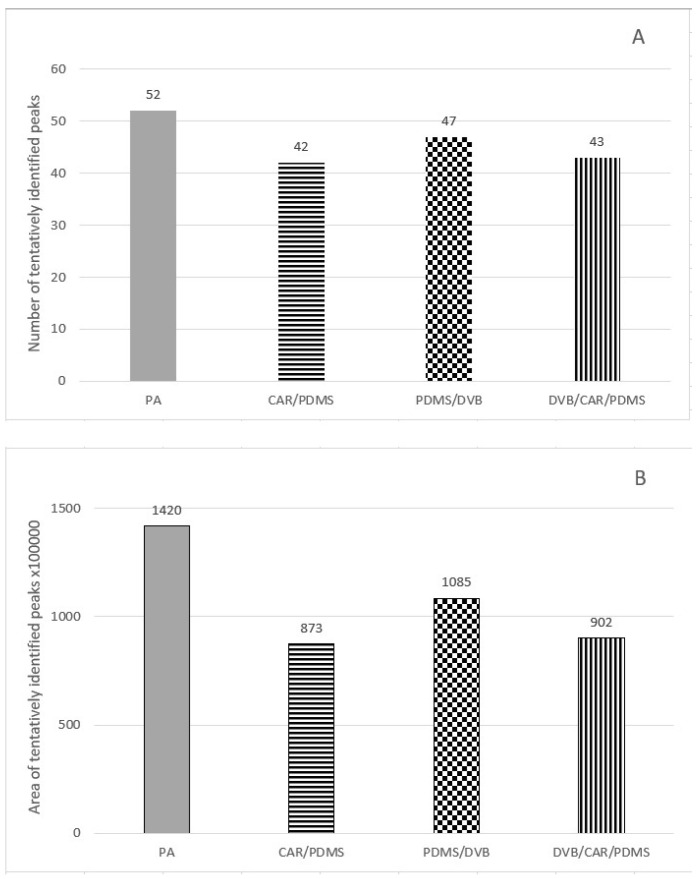
Comparison of the extraction efficiency of volatile compounds from Rondo wine by HS-SPME using different fibers under standard extraction conditions. Wine volume—3 mL in a 7 mL vial (undiluted wine); addition of NaCl—0.9 g; addition of diluted HCl—50 μL; addition of internal standard—100 μL; stirring speed—minimum; temperature of sample incubation—40 °C; time of sample incubation—15 min; temperature of extraction—40 °C; time of extraction—30 min. The results are expressed as (**A**) number of tentatively identified peaks and (**B**) area of tentatively identified peaks.

**Figure 2:**
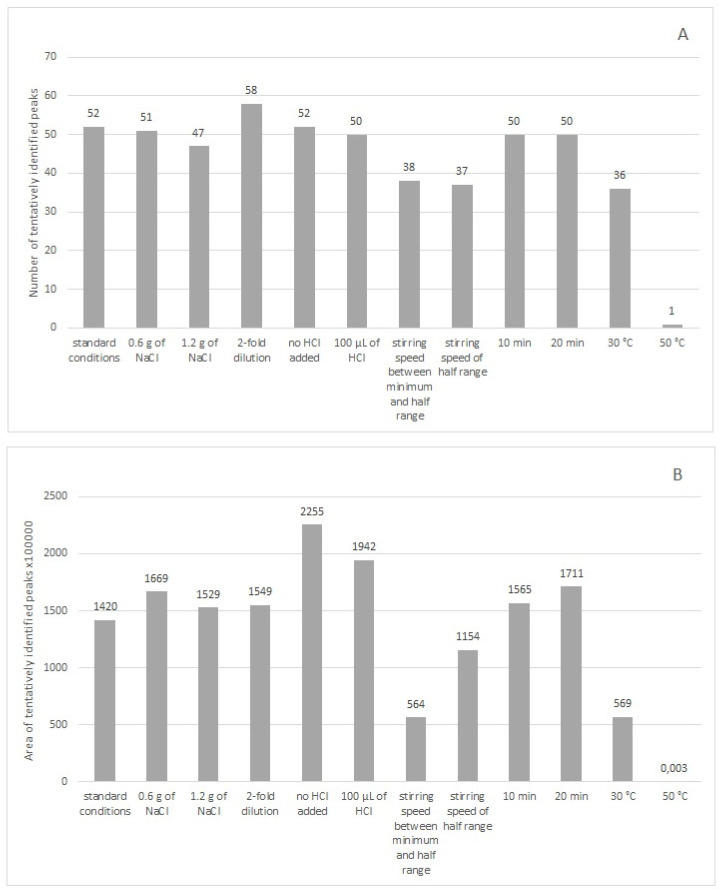
Comparison of the extraction efficiency of volatile compounds from Rondo wine (R2) by HS-SPME using a PA fiber under different conditions. The results are expressed as (**A**) number of tentatively identified peaks and (**B**) area of tentatively identified peaks.

Initially, extractions were performed on four different fibers, polyacrylate (PA), carboxen-polydimethylosiloxane (CAR/PDMS), polydimethylosiloxane-divinylbenzene (PDMS/DVB) and divinylbenzene-carboxen-polydimethylosiloxane (DVB/CAR/PDMS), under the same, standard conditions in order to select the fiber that allowed the obtention of the highest number of tentatively identified chromatographic peaks. This is of particular importance when a marker of wine adulteration is sought. Standard extraction conditions for all the fibers were as follows: 3 mL of wine (undiluted) in a 7 mL vial, 0.9 g of NaCl, 50 μL of diluted HCl, 100 μL of internal standard, minimum stirring speed, incubation temperature 40 °C, incubation time 15 min, extraction temperature 40 °C and extraction time 30 min. Rondo wine (R2) was used for the fiber selection. Comparison of the extraction efficiency of volatile compounds from Rondo wine by HS-SPME using different fibers under standard extraction conditions is shown in Figure 1. The largest number of tentatively identified peaks were extracted on the PA fiber, and the area of these peaks was the largest among all the fibers. Thus, the optimization of the extraction conditions was performed on the PA fiber. Rondo wine (R2) was used for optimization. The following parameters were optimized: addition of NaCl (0.6 g; 1.2 g), wine dilution with water (2-fold dilution), addition of diluted HCl (no addition; 100 μL), stirring speed (between minimum and half range; half range), extraction temperature (30 °C; 50 °C) and extraction time (10 min; 20 min). In successive extractions, one parameter of standard extraction conditions was changed, leaving the other parameters unchanged. Comparison of the extraction efficiency of volatile compounds under different conditions is shown in Figure 2. Fifty-two tentatively identified compounds were extracted under standard conditions. The largest number of tentatively identified compounds (58) was extracted when wine was 2-fold diluted. Although the volatiles extracted without the addition of HCl had higher area value than those extracted with 2-fold dilution, we chose 2-fold dilution as optimal to extract as many compounds as possible. Thus, the optimal extraction conditions for PA fiber were as follows: 1.5 mL of wine and 1.5 mL of distilled water in a 7 mL vial (2-fold dilution), 50 μL of diluted HCl, 100 μL of internal standard, minimum stirring speed, incubation temperature 40 °C, incubation time 15 min, extraction temperature 40 °C and extraction time 30 min. In summation, the optimal conditions for PA fiber differed from the standard extraction conditions in that a 2-fold dilution of wine was used.

Sixty-seven volatile compounds were tentatively identified in the Zweigelt and Rondo wines; they are presented in Table 1. These compounds represent several classes: acids (9 compounds), alcohols (24 compounds), aldehydes (2 compounds), esters (19 compounds), furan compounds (2 compounds), ketones (2 compounds), sulfur compounds (1 compound) and terpenes (8 compounds). The relative concentrations of volatile compounds in the wines produced from grapes of the Zweigelt and Rondo varieties are shown in Tables S1 and S2 in the Supplementary Material, respectively. Also, chromatograms of the volatile compounds of Zweigelt and Rondo wines are shown in Figures S1 and S2 in the Supplementary Material, respectively. To verify whether the tested wines differed in the proportions of aroma compound classes, the subtotal concentration of the particular classes and their percentage share in the total content of volatile compounds were calculated. The proportions of volatile compounds in Zweigelt and Rondo wines were similar. A majority of the aroma compounds were alcohols considering their number and the concentration of volatiles identified in the wines. The concentrations of alcohols were 67.28–80.10% of the total volatile compounds in Zweigelt wines and 67.12–87.78% in Rondo wines. Both Zweigelt and Rondo wines had average concentrations of esters and acids. The concentrations of esters ranged from 6.88% to 17.42% in Zweigelt wines, and from 5.25% to 18.03% in Rondo wines, and acids ranged from 6.69% to 12.73% and from 4.29% to 11.39%, respectively. The minor compounds were ketones, terpenes, aldehydes, sulfur compounds and furan compounds.

The total concentration of volatile compounds tentatively identified in Zweigelt wines ranged from 1969.81 μg/L to 4260.79 μg/L (Table S1). The Z3 wine had the lowest concentration of volatile compounds—Z5 had the highest. The lowest subtotal concentration of alcohols was found in Z3 (1325.32 μg/L) and the highest in Z5 (3412.74 μg/L). The dominant alcohols in the Zweigelt wines were 3-methylbutan-1-ol, 2-phenylethanol and 2-methylpropan-1-ol. The subtotal concentration of esters ranged from 181.31 μg/L in Z4 to 472.89 μg/L in Z1LAB. This volatile fraction was mainly composed of ethyl 2-hydroxypropanoate, ethyl octanoate and diethyl butanedioate. We did not identify ethyl acetate, unlike Jurek [37]. The subtotal concentration of acids varied from 202.631 μg/L in Z3 to 358.72 μg/L in Z5, and the major acids were octanoic, hexanoic and acetic acid. Zweigelt wines contained between 62.25 μg/L and 139.32 μg/L of ketones in Z4LAB and Z1, respectively. Of the two ketones tentatively identified in those wines, 4-methyl-3-penten-2-one was the more abundant one. The subtotal concentration of terpenes ranged from 1.99 μg/L in Z1LAB to 55.05 μg/L in Z1. Among the tentatively identified terpenes, (*E*)-6,10-dimethyl-5,9-undecadien-2-one (geranylacetone) occurred at the highest relative concentrations, followed by (*E*)-1-(2,6,6-trimethyl-1,3-cyclohexadien-1-yl)-2-buten-1-one (β-damascenone) and 1,1,6-trimethyl-1,2-dihydronaphthalene (TDN). The subtotal concentration of aldehydes varied from 0.60 μg/L in Z2 to 15.26 μg/L in Z5. Benzaldehyde was the more abundant compound of the two detected aldehydes. The relative content of the only sulfur compound (3-(methylsulfanyl) propan-1-ol) tentatively identified in this present study ranged from 0.66 μg/L in Z2 LAB to 1.63 μg/L in Z1LAB. Finally, the relative concentrations of the two furan compounds varied from 0.37 μg/L in Z3 to 1.61 μg/L in Z4LAB.

The relative concentrations of total volatile compounds in the Rondo wines ranged from 1986.35 μg/L in R3LAB to 3846.62 μg/L in R5 (Table S2). The subtotal concentration ranged from 1333.15 to 3273.99 μg/L for alcohols, 147.90–527.25 μg/L for esters, 130.02–250.04 μg/L for acids, 45.79–106.65 μg/L for ketones, 1.21–56.21 μg/L for terpenes, 3.62–6.81 μg/L for aldehydes, 0.34–5.98 μg/L for furan compounds and 1.51–3.11 μg/L for the sulfur compound. The Rondo wines were characterized by a high relative contents of the alcohols 3-methylbutan-1-ol, 2-phenylethanol and 2-methylpropan-1-ol, the esters ethyl 2-hydroxypropanoate, ethyl octanoate and diethyl butanedioate, octanoic, acetic and hexanoic acids, the ketone 4-methyl-3-penten-2-one, the terpenes (*E*)-6,10-dimethyl-5,9-undecadien-2-one (geranylacetone) and (*Z*)-linalool oxide, as well as benzaldehyde and dihydrofuran-2(3H)-one. The main compounds in individual classes of volatiles were the same as in our previous papers [38,39,40] with the exception of octanoic acid. This compound was the main acid in the present study, while acetic acid was reported as the most abundant acid in our previous papers [38,40]. 3-(Methylsulfanyl) propan-1-ol was the only sulfur compound tentatively identified in this study, which is in agreement with our previous papers [38,40] and in contrast with work by Liu et al. [41], in which 2-methyldihydro-3(2H)-thiophenone was the only sulfur compound identified.

When comparing all the test wines produced from grapes of the Zweigelt and Rondo varieties, we found that some of the Zweigelt wines did not contain some of the compounds tentatively identified in all Rondo wines and vice versa. Some Zweigelt wines did not contain benzoic acid, octen-3-ol and benzaldehyde, while some Rondo wines did not contain 2-methylpropanoic acid, butan-1-ol, ethyl benzoate, ethyl 9-decenoate, (*Z*)-linalool oxide and 3,7-dimethyl-1,6-octadien-3-ol (β-linalool). One of the volatile compounds tentatively identified in this study—3,7-dimethyl-1,5,7-octatrien-3-ol (hotrienol)—was detected in all Zweigelt wines but was not present in the Rondo wines at all. This means that hotrienol can be used as a marker for Zweigelt wines. Hotrienol belongs to the class of terpenes. Jurek [38] identified this terpene as well as several other terpenes, such as linalool, α-terpineol, citronellol, vitispiran and TDN, in Zweigelt wines.

Wines produced from the same grape variety, Zweigelt or Rondo (Z1–Z5 or R1–R5), in which AF was carried out by different commercial yeast strains and MLF was spontaneous, differed in the relative concentrations of individual volatile compounds. Furthermore, among Z1–Z5 wines, only Z1 contained (*E*)-6,10-dimethyl-5,9-undecadien-2-one (geranylacetone) and among R1–R5 wines, only R2 contained (*Z*)-linalool oxide. Thus, the yeast strain influenced the content of these compounds. Similarly, Gammacurta et al. [42] showed that the concentrations of esters in Cabernet Sauvignon wines from the Bordeaux region depended on the commercial strain of *S. cerevisiae* used. Moreover, Liu et al. [43] found that volatile compounds levels, including terpene levels, were contingent on yeast strains. The different levels of terpenes may have been a product of the activity of β-glucosidase secreted by yeast and releasing monoterpene alcohol from the bound terpenoid precursor. However, several terpenoids can also be synthesized by *S. cerevisiae* by the de novo pathway. Additionally, the production of terpene alcohols was related to other reactions, such as chemical isomerization, hydration or reduction, conducted by wine yeasts [44].

In the case of Zweigelt wines subjected to different types of MLF, i.e., spontaneous MLF (Z1–Z5) or induced MLF (Z1 LAB–Z5 LAB), the relative contents of 2-phenylethyl acetate and dihydrofuran-2(3H)-one (butyrolactone) were lower in the former compared to the latter wines. On the other hand, the relative contents of diethyl-2-hydroxybutanedioate and methyl hexadecanoate were higher in the Z1–Z5 wines compared to the Z1LAB–Z5LAB wines. As for Rondo wines subjected to spontaneous MLF (R1–R5) and induced MLF (R1 LAB–R5 LAB), the relative contents of ethyl 2-hydroxypropanoate (ethyl lactate), 2-methylpropyl 2-hydroxypropanoate (isobutyl lactate), 3-methylbutyl 2-hydroxypropanoate (isoamyl lactate), 2-phenylethyl acetate and dihydrofuran-2(3H)-one were lower in the former compared to the latter wines. However, the contents of ethyl benzoate, ethyl hexadecanoate and (*E*)-1-(2,6,6-trimethyl-1,3-cyclohexadien-1-yl)-2-buten-1-one (β-damascenone) were higher in R1–R5 than in R1LAB–5LAB. Wines R1–R5 contained diethyl-2-hydroxybutanedioate (diethyl malate), while R1LAB–5LAB did not contain this compound. Our results regarding the relative content of ethyl lactate in wines subjected to different types of MLF are in agreement with the results of Abrahamse and Bartowsky [45], Costello et al. [46], Lasik-Kurdyś et al. [47], Malherbe et al. [48] and our previous research [38]. All these authors state that ethyl lactate is a characteristic volatile product of MLF. Also, the results of the analysis of butyrolactone and 2-phenylethyl acetate conducted in this present study are similar to our previous findings [38]. In contrast to our study, Abrahamse and Bartowsky [45] reported that the concentration of 2-phenylethyl acetate was higher in wine subjected to spontaneous MLF compared to induced MLF. On the other hand, Costello et al. [46], Lasik-Kurdyś et al. [47] and Malherbe et al. [48] found that the type of MLF had no significant effect on the content of 2-phenylethyl acetate.

**Figure 3:**
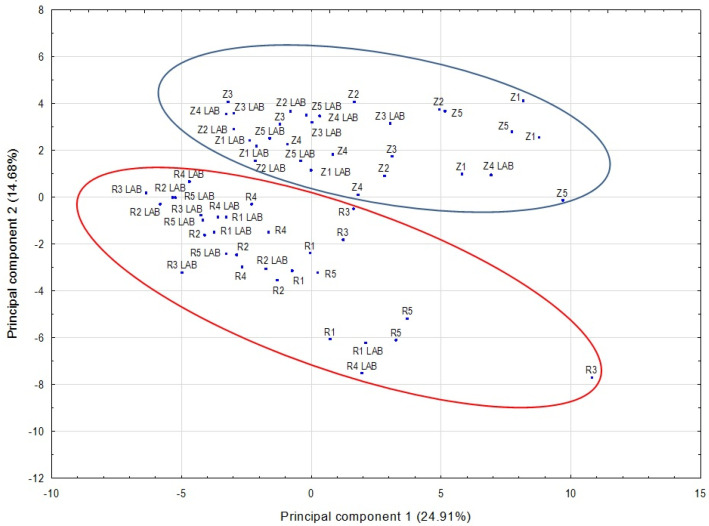
Score plot on the PCA plane defined by the first two principal components for all volatile compounds found in the wine samples. Z1–Z5—Zweigelt wines in which AF was induced using various yeast strains and MLF was spontaneous. Z1 LAB–Z5 LAB—Zweigelt wines in which AF was induced using various yeast strains (the same strains as in Z1–Z5 wines) and MLF was carried out by inoculation with lactic acid bacteria. R1–R5—Rondo wines in which AF was induced using various yeast strains and MLF was spontaneous. R1 LAB–R5 LAB—Rondo wines in which AF was induced using various yeast strains (the same strains as in R1–R5 wines) and MLF was carried out by inoculation with lactic acid bacteria.

**Figure 4:**
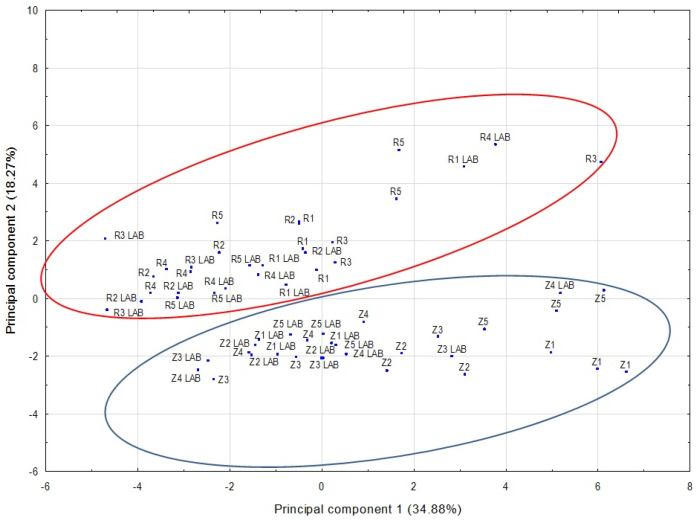
Score plot on the PCA plane defined by the first two principal components for alcohols found in the wine samples. Z1–Z5—Zweigelt wines in which AF was induced using various yeast strains and MLF was spontaneous. Z1 LAB–Z5 LAB—Zweigelt wines in which AF was induced using various yeast strains (the same strains as in Z1–Z5 wines) and MLF was carried out by inoculation with lactic acid bacteria. R1–R5—Rondo wines in which AF was induced using various yeast strains and MLF was spontaneous. R1 LAB–R5 LAB—Rondo wines in which AF was induced using various yeast strains (the same strains as in R1–R5 wines) and MLF was carried out by inoculation with lactic acid bacteria.

In our study, PCA was used for preliminary data analysis to visualize the potential grouping of samples. PCA is based on a linear transformation of data into a set of new orthogonal variables called principal components [10,16,21,49]. The PCA for all the compounds revealed that, according to the Kaiser–Guttman criterion, the first 14 principal components had eigenvalues greater than 1 and this explained 85.85% of the total variance. Analyzing the location of the points representing the data on the plane formed by the first two principal components, we found that grape variety may be a factor differentiating the wines (Figure 3). However, the two principal components explained only 39.59% of the variance. A PCA run for alcohols showed that the first 6 principal components had eigenvalues greater than 1 and explained 77.33% of the total variance. An analysis of the wines on a plane spanning the first two principal components indicated that the wines formed two varietal groups (Figure 4). The first two principal components explained 53.15% of the total variance. PCA for the other classes of volatile compounds, acids, esters, terpenes and others (aldehydes, furan compounds, ketones and the sulfur compound), did not indicate that the wines could be grouped by variety.

Although PCA allowed us to separate the two grape varieties for wine production in the case of all the investigated compounds and alcohols, the first two principal components failed to explain the variance sufficiently. Therefore, alternative data treatment methods, SVM and kNN, were used. The advantage of using these machine learning techniques is that they do not require any assumptions to be made about data distribution or homogeneity of variances [49,50].

SVM is a supervised technique whose aim is to find a hyperplane in a p-dimensional space (p is the number of variables) to separate classes of data [49,50]. The major advantage of SVM is that the learning capacity is good enough even if there are many features. To separate two classes of data, several possible hyperplanes could be chosen [15]. In this study, we implemented SVM using a radial basis function (RBF) kernel. Table 2 shows the classification accuracy of SVM depending on the relative content of the investigated volatile compounds in the test set and by variety. Using SVM for all the test compounds or separately for alcohols only, we obtained models with the highest possible classification accuracy (100%). When acids alone were considered, it was possible to classify the wines in the test set at an accuracy of 93.33%, with Rondo wines classified at 87.5% accuracy and Zweigelt wines classified at 100% accuracy. When terpenes only were taken into account, the classification accuracy was 93.33% in the test set; Rondo wines were classified at 100% accuracy and Zweigelt wines at 83.3% accuracy. The weakest classification accuracy (86.67%) was achieved for wines using models built on the basis of the seven compounds from the ‘others’ group and esters. Overall, SVM provided a more accurate classification of the Zweigelt wines than of the Rondo wines. The wines of the Zweigelt variety were classified at 100% accuracy by analyzing the relative content of all compounds, acids alone, alcohols alone and esters alone. A 100% classification accuracy for the Rondo wines was achieved when we analyzed all compounds, alcohols alone and terpenes alone.

kNN is a supervised machine learning technique mainly used for classification problems. This technique consists in classifying a data point by analyzing the nearest data points. The advantage of kNN is that the use of the same or a very similar number of samples for the analyzed classes results in a reliable classification [15]. In this study, kNN was implemented with a Euclidean distance measure between data points. Table 3 presents the classification accuracy of KNN in the test set and by variety with the optimal number of k nearest neighbors. When all the test compounds or alcohols alone were considered, kNN allowed the obtention of models with the highest possible classification accuracy (100%). When esters only were taken into account, the classification accuracy was 93.33% in the test set, with wines of the Rondo variety classified at 100% accuracy and the Zweigelt wines at 88.89% accuracy. For terpenes, the classification accuracy was 93.33% in the test set; Rondo and Zweigelt wines were classified at 90% and 100% accuracy, respectively. Again, the model obtained on the basis of the compounds belonging to the ‘others’ group provided the weakest classification accuracy (80%). Similarly to SVM, kNN provided a more accurate classification of the Zweigelt wines than of the Rondo wines. The Zweigelt wines were classified 100% correctly when the relative contents of all compounds, alcohols only, terpenes only and others only were analyzed; 100% correct classification of Rondo wines was achieved when we considered all the compounds, or alcohols only or esters only.

In this study, the relative concentrations of all volatile compounds or alcohols alone were used as an input data set for the successful varietal authentication of Polish wines by SVM and kNN. In the past, Gómez-Meire et al. [18] investigated the suitability of applying a semi-quantitative analysis of volatiles and different machine learning techniques, such as SVM, RF, MLP, kNN and NB, for the classification of Spanish wines from four autochthonous white grape varieties (Albariño, Treixadura, Loureira and Dona Branca). The authors obtained perfect classification by the RF algorithm using all the volatiles determined in the wines, while the other techniques yielded promising results using only some classes of volatile compounds. Moreover, some authors had used modeling of GC-MS fingerprints. Majchrzak et al. [16] applied SVM to classify white and red wines according to the variety and obtained an accuracy of 98.7 and 98.2%, respectively. On the other hand, Springer [17] used a decision tree for the authentication of similar grape varieties for wine production and achieved 85–98% correct classification of external samples.

**Figure 5:**
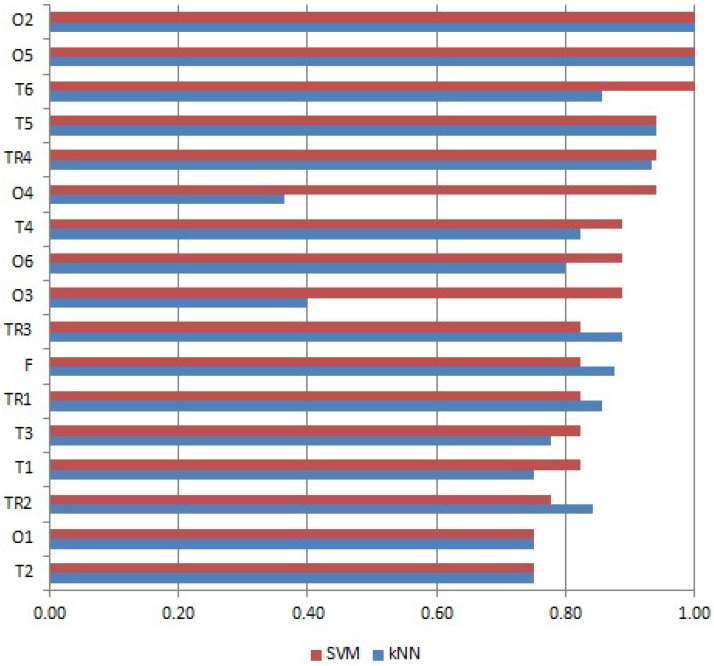
F-score ranking of importance of subsets formed from selected compounds obtained with the use of SVM and kNN methods (see Table 4 for notations).

According to Costa et al. [51], the variable selection method allows to reduce computation time, improve prediction and better understand the data in machine learning methods. It is possible to simplify the classification model by eliminating redundant or irrelevant variables from the data set. A nonparametric Mann–Whitney test was used to verify whether the wines made from Zweigelt and Rondo varieties differed in the content of the studied compounds. Based on the results, the number of variables was reduced to 37. In the next step, in order to further reduce the number of variables, after analyzing the *p*-value (*p*-value < 0.0000001 in the Mann–Whitney test, Table S3 in the Supplementary Materials), descriptive statistics (mean, maximum, minimum and variance) of the tested compounds and the values of factor loadings in the PCA analysis (variables with the highest factor loadings given by PCA for the first two principal components, Figure S3 in the Supplementary Materials), six of the compounds with the highest potential to distinguish between Zweigelt and Rondo wines were selected: 3-ethyl-4-methylpentan-1-ol, octen-3-ol, butane-2,3-diol, 2-phenylethyl acetate, 3,7-dimethyl-1,5,7-octatrien-3-ol (hotrienol) and 3-(methylsulfanyl)propan-1-ol. Then, one-, two-, three-, four-, and five-variable subsets of the selected compounds were constructed. SVM and kNN were applied to the subsets as well as to the entire set of the selected compounds. The importance value of these subsets was evaluated on the basis of the F-score. It was revealed that SVM and kNN methods yielded the best classification models (F-score of 1 and accuracy of 100%) when 3-ethyl-4-methylpentan-1-ol or 3,7-dimethyl-1,5,7-octatrien-3-ol (hotrienol) or subsets containing one or both of them were taken into account. This was due to two facts. Firstly, Zweigelt wines were characterized by a much higher relative content of 3-ethyl-4-methylpentan-1-ol, an average of 1.21 μg/L (min = 0.67, max = 1.96), than Rondo wines, which on average contained 0.18 μg/L (min = 0, max = 0.39) of this compound, and secondly, 3,7-dimethyl-1,5,7-octatrien-3-ol (hotrienol) was not present in the Rondo wines at all but was detected in all samples of the Zweigelt wine at an average concentration of 0.60 μg/L (min = 0.27, max = 1.31). The accuracy of classification and F-score values are shown in Table 4 and Figure 5, respectively. The subsets of two or more variables containing 3-ethyl-4-methylpentan-1-ol or 3,7-dimethyl-1,5,7-octatrien-3-ol (hotrienol) were omitted to simplify the presentation. Moreover, the best model (F-score of 1) was built with subset T6 containing 2-phenylethyl acetate and 3-(methylsulfanyl)propan-1-ol for SVM because Zweigelt and Rondo wines differed in the relative concentrations of these compounds. The average concentration of 2-phenylethyl acetate in Zweigelt wines was 1.12 μg/L (min = 0, max = 4.37) while in Rondo wines it was 4.71 μg/L (min = 0, max = 12.38). In turn, the average concentration of 3-(methylsulfanyl)propan-1-ol in Zweigelt wines was 1.11 μg/L (min = 0.54, max = 1.70) and in Rondo wines was 2.20 μg/L (min = 1.08, max = 3.89). The worst models (F-score of 0.75) were built with subsets O1 and T2 (accuracy of 73.33%) using SVM. The worst kNN model (F-score equal 0.36) was obtained for 2-phenylethyl acetate (accuracy of 53.33%).

## 3. Materials and Methods

### 3.1. Winemaking Process and Wine Samples

The details of the winemaking process are presented in our previous article [26]. The grapes of the Zweigelt and Rondo varieties originated from ‘Małe Dobre’ and ‘Dom Bliskowice’ vineyards, respectively. The vineyards are located in the Lublin Province, Poland. The parameters of grape musts used for fermentation were as follows: Zweigelt must—extract value 22 Blg, pH 3.31, total acidity as tartaric acid 6 g/L; Rondo must—extract value 20 Blg, pH 3.61, total acidity as tartaric acid 6.45 g/L. Alcoholic fermentation (AF) was performed using five commercial yeast strains: four *Saccharomyces cerevisiae* (SafOEno TM SC 22, Essentiale Grand Cru (Lesaffre, France), Siha Active Yeast 8 and Siha Rubino Cru (Eaton, Tinton Falls, NJ, USA)) and one *S. cerevisiae x S. bayanus* (SafOEno TM HD S62 (Lesaffre, France)) for both Zweigelt and Rondo wines. One part of the wines was left to undergo spontaneous MLF without inoculation with lactic acid bacteria (LAB), and the remaining parts were subjected to MLF by inoculation with the LAB *Oenococcus oeni*—Viniflora Oenos (Eaton, Tinton Falls, NJ, USA). *O. oeni* starter culture was added after the completion of AF (sequential inoculation) to the part of wines in which MLF was induced. The winemaking process was performed in duplicate. The parameters of the final wines, such as individual sugars, acids, pH and total acidity, are presented in the supplementary material to our previous article [26]. Table 5 presents a description of wines. Twenty different wines were produced from October 2017 to April 2018 and analyzed in April 2019. Three bottles of each wine were taken for analysis (60 samples in total).

### 3.2. Chemicals

Sodium chloride and hydrochloric acid were obtained from POCh (Gliwice, Poland). Sodium chloride was oven dried at 200 °C overnight. Hydrochloric acid (37%) was dissolved in water at a concentration of 78 g/L. 4-Hydroxy-4-methyl-2-pentanone (the internal standard) was purchased from Sigma-Aldrich (Saint Louis, MO, USA) and prepared in water at a concentration of 7 mg/L. A mixture of n-alkanes (C7–C30) for the calculations of linear temperature programmed retention indices (LTPRI) was supplied by Supelco (Bellefonte, PA, USA). All chemicals were of an analytical grade.

### 3.3. SPME-GC/MS

#### 3.3.1. HS-SPME

A fiber for the extraction of wines was selected from the following fibers: PA, CAR/PDMS, PDMS/DVB and DVB/CAR/PDMS (Supelco, Bellefonte PA, USA). The fibers were preconditioned according to the manufacturer’s instructions. Standard extraction conditions for all the fibers were as follows: in a glass vial of 7 mL, 0.9 g of NaCl, 3 mL of wine (undiluted), 50 μL of diluted HCl, 100 μL of 4-hydroxy-4-methyl-2-pentanone (as an internal standard) and a magnetic stirring bar was placed. Rondo wine (R2) was used for the fiber selection. The vial was tightly capped with a polytetrafluoroethylene (PTFE)-silicone septum (Supelco, Bellefonte, PA, USA), on which a screw cap with a hole was placed. The vial was placed on an MS7-H550-S hotplate magnetic stirrer (DLAB Scientific Co., Beijing, China) in a block to ensure uniform heat distribution. Each wine sample was incubated at 40 °C for 15 min under continuous stirring at a minimum speed prior to extraction. Then, the fibers were exposed to the headspace (HS) at 40 °C for 30 min under continuous stirring. After extraction, the fiber was removed from the vial and thermally desorbed in the GC injection port for 2 min at 220 °C in split-less mode. Prior to each analysis, the fiber was cleaned by inserting into the auxiliary GC injection port at 280 °C for 5 min.

SPME conditions were optimized on PA fiber. Rondo wine (R2) was used for optimization. The following parameters were optimized: addition of NaCl (0.6 g; 1.2 g), wine dilution with water (2-fold dilution), addition of diluted HCl (no addition; 100 μL), stirring speed (between minimum and half range; half range), extraction temperature (30 °C; 50 °C) and extraction time (10 min; 20 min). In successive extractions, one parameter of standard extraction conditions was changed, leaving the other parameters unchanged. The optimal extraction conditions for PA fiber were as follows: 0.9 g of NaCl, 1.5 mL of wine, 1.5 mL of distilled water, 50 μL of diluted HCl, 100 μL of 4-hydroxy-4-methyl-2-pentanone, incubation at 40 °C for 15 min under continuous stirring at a minimum speed prior to extraction and exposition to the headspace (HS) at 40 °C for 30 min under continuous stirring. All wines were extracted under optimal conditions.

#### 3.3.2. GC/MS

The wines were analyzed in triplicate using a GCMS-QP2010 gas chromatograph coupled to a quadrupole mass spectrometer (Shimadzu, Kyoto, Japan). The chromatographic technique was presented in our previous publication [38]. Chromatographic separations were carried out using a VF-WAXms capillary column with the following characteristics: 60 m, 0.25 mm ID × 0.25 μm film thickness and 100% polyethylene glycol (Agilent, Santa Clara, CA, USA). The carrier gas was helium at a flow rate of 1.8 mL/min. The column oven temperature program was as follows: initial temperature 34 °C for 5 min, 34–100 °C at a rate of 3 °C/min and held for 6 min and 100–220 °C at a rate of 5 °C/min and held for 15 min. The total run time was 72 min. An electron ionization source was used with a source temperature of 200 °C and an electron energy of 70 eV. Mass spectral data were collected over the range of m/z 30–300 in the full scan mode (scan time 0.4 s). Data were acquired using GCMSsolution software version 2. Volatile compounds were tentatively identified on the basis of their mass spectra and experimental LTPRI. Mass spectrometric information of each chromatographic peak was compared to the NIST 05 mass spectral library, considering a minimum similarity value of 80%. A mixture of n-alkanes (C7–C30) diluted in hexane (Supelco, Bellefonte, PA, USA) was loaded onto the SPME fiber and injected under the temperature program mentioned earlier in this subsection to calculate experimental LTPRI of each extracted compound. Experimental LTPRIs were compared to the retention indices reported in the literature for similar chromatographic columns. Semi-quantitative data of the aroma compounds were calculated by dividing the peak area of a compound with the peak area of the internal standard and multiplying the result with the concentration of the internal standard (233.33 µg/L). The concentrations of the volatiles were expressed as μg/L.

### 3.4. Statistical Analysis

**Figure 6:**
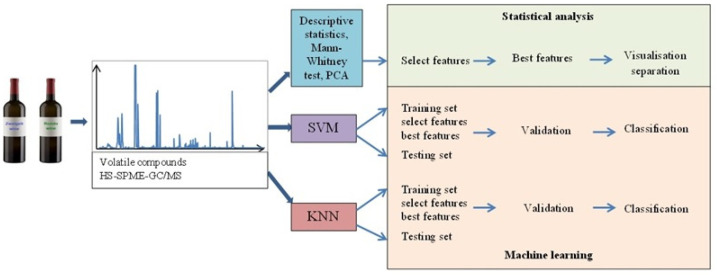
Methodology used for the analysis of Zweigelt and Rondo wines.

The data were analyzed statistically using the Statistica 13.3 software package (Statsoft, Krakow, Poland) and open-source software Python 3.7, library: Scikit-learn. All the test compounds were analyzed and divided into groups: acids, alcohols, esters, terpenes and others (the group “others” included aldehydes, furan compounds, ketones and sulfur compound). PCA was used for preliminary data analysis to visualize the potential grouping of samples. In PCA, significant principal components were selected based on the Kaiser–Guttman criterion (principal components whose eigenvalues were greater than 1 were chosen). The SVM and kNN machine learning techniques were used for data classification. SVM using a radial basis function (RBF) kernel and kNN with a Euclidean distance measure between data points were implemented. The dataset was not particularly large, so the models in both SVN and kNN were firstly verified by 10-fold cross-validation. Next, the data set was randomly divided into two subsets, training and testing, which contained 75% and 25% of observations, respectively. SVM and kNN techniques were applied. An RBF kernel in the SVM method has two parameters, which must be tuned to achieve a good performance. A grid search of these parameters for the kernel was performed using GridSearchCV function in Python. Then, the F-score was used to generate a ranking of importance for subsets formed from the selected variables. The methodology used in the study is summarized in Figure 6.

## 4. Conclusions

In this paper, we found that the compound 3,7-dimethyl-1,5,7-octatrien-3-ol (hotrienol) could be used as a variety marker to distinguish Zweigelt wines from Rondo wines as it does not occur in the latter. Furthermore, we proposed classification models of red wines produced from two grape varieties, i.e., Zweigelt and Rondo, for the assessment of varietal authenticity. For the first time, the relative concentrations of volatile compounds were used for the varietal authentication of wines produced in Poland.

PCA allowed us to separate Zweigelt and Rondo wines in the case of all the test compounds and alcohols, but the first two principal components failed to explain the variance sufficiently. Wines were classified according to grape variety at 100% accuracy by the machine learning methods SVM and kNN, although wines from the same grape variety had different relative concentrations of the individual volatile compounds because they were produced using different yeast strains and types of malolactic fermentation. Application of the variable selection method simplified the classification model. The most important variables were 3-ethyl-4-methylpentan-1-ol and 3,7-dimethyl-1,5,7-octatrien-3-ol (hotrienol) for SVM and kNN as well as 2-phenylethyl acetate and 3-(methylsulfanyl)propan-1-ol for SVM.

The state-of-the-art approach to varietal authentication presented in this paper can be applied in the control of wine quality. The classification models may be expanded to include wines from other grape varieties differing in production methods. The relative concentrations of volatile compounds in the Zweigelt and Rondo wines can be used to create databases of authentic wines. Further research on Zweigelt and Rondo wines produced in different vineyards located in different geographical regions of Poland is needed to show whether the wines can be classified independently of the region of origin.

## Figures and Tables

**Table 1:** Volatile compounds identified in Zweigelt and Rondo wines.

Peak No.	Compound	Similarity (%)	RT (min)	LTPRI Exp.	LTPRI Lit.	References
	Acids					
20	Acetic acid	99	34.874	1469	1457	[27]
28	Propanoic acid	89	39.088	1554	1536	[27]
31	2-Methylpropanoic acid	98	40.221	1580	1573	[28]
52	Hexanoic acid	98	48.552	1852	1851	[28]
59	Octanoic Acid	98	53.311	2061	2067	[28]
60	Nonanoic acid	94	55.426	2167	2170	[27]
63	Decanoic acid	98	57.416	2270	2281	[28]
66	Benzoic acid	78	61.369	2449	2434	[29]
67	Dodecanoic acid	93	62.125	2479	2488	[28]
	Alcohols					
2	2-Methylpropan-1-ol	98	16.232	1121	1100	[30]
4	Butan-1-ol	97	18,828	1171	1173	[27]
5	3-Methylbutan-1-ol	99	21.775	1228	1221	[31]
6	Pentan-1-ol	95	23.532	1263	1259	[28]
8	4-Methylpentan-1-ol	96	26.403	1319	1309	[32]
9	3-Methylpentan-1-ol	98	26.989	1330	1322	[32]
11	Hexan-1-ol	98	28.237	1353	1361	[28]
12	(*E*)-3-Hexen-1-ol	96	28.867	1365	1358	[32]
13	3-Ethoxypropan-1-ol	93	29.520	1378	1371	[27]
14	(*Z*)-3-Hexen-1-ol	90	29.925	1386	1379	[32]
18	Octen-3-ol	96	33.955	1454	1451	[33]
19	Heptan-1-ol	97	34.282	1459	1470	[27]
22	2-Ethylhexan-1-ol	98	36.215	1491	1486	[32]
23	3-Ethyl-4-methylpentan-1-ol	92	37.176	1509	1509	[27]
26	Butane-2,3-diol	98	38.732	1545	1563	[27]
29	Octan-1-ol	99	39.344	1560	1567	[28]
32	Propane-1,2-diol	90	40.855	1595	1591	[27]
34	2-(2-Ethoxyethoxy)- ethanol	96	41.732	1620	1622	[27]
39	Nonan-1-ol	97	43.083	1660	1656	[29]
46	Decan-1-ol	95	46.147	1761	1755	[29]
54	Phenylmethanol	80	49.320	1883	1879	[34]
56	2-Phenylethanol	97	50.168	1919	1919	[28]
57	Dodecan-1-ol	97	51.181	1963	1959	[29]
65	Hexadecan-1-ol	96	59.427	2368	2400	[33]
	Aldehydes					
24	Benzaldehyde	92	37.892	1526	1522	[28]
38	4-Methylbenzaldehyde	92	42.774	1651	1638	[32]
	Esters					
1	Ethyl 3-methylbutanoate	93	14.443	1079	1066	[32]
10	Ethyl 2-hydroxypropanoate	98	27.943	1348	1338	[27]
15	Methyl octanoate	87	29.967	1386	1381	[27]
16	Ethyl octanoate	98	32.775	1434	1429	[27]
21	2-Methylpropyl 2-hydroxypropanoate	94	34.665	1465	1454	[35]
25	Ethyl nonanoate	91	38.340	1536	1540	[28]
30	3-Methylbutyl 2-hydroxypropanoate	98	39.889	1572	1568	[35]
37	Ethyl decanoate	95	42.336	1638	1643	[27]
40	Ethyl benzoate	87	43.403	1669	1665	[27]
41	Diethyl butanedioate	96	43.675	1677	1672	[32]
42	Ethyl 9-decenoate	92	44.067	1689	1697	[28]
47	Methyl 2-hydroxy benzoate	82	46.712	1781	1775	[27]
48	Ethyl phenylacetate	95	46.937	1789	1787	[28]
49	2-Phenylethyl acetate	97	47.730	1819	1810	[32]
51	Ethyl dodecanoate	91	48.281	1841	1840	[29]
55	Ethyl 3-phenylpropanoate	92	49.464	1889	1892	[27]
58	Diethyl-2-hydroxybutanedioate	89	52.939	2044	2038	[27]
61	Methyl hexadecanoate	92	56.302	2212	2211	[29]
62	Ethyl hexadecanoate	84	56.970	2247	2243	[27]
	Furan compounds					
35	Ethyl 2-furoate	87	41.936	1626	1627	[27]
36	Dihydrofuran-2(3H)-one	89	42.283	1636	1627	[28]
	Ketones					
3	4-Methyl-3-penten-2-one	98	17.497	1145	1139	[36]
7	3-Hydroxybutan-2-one	93	25.403	1300	1289	[28]
	Sulphur compounds					
44	3-(Methylsulfanyl)propan-1-ol	96	45.001	1720	1715	[32]
	Terpenes					
17	(*Z*)-Linalool oxide	96	33.397	1444	1446	[29]
27	3,7-Dimethyl-1,6-octadien-3-ol (β-Linalol)	93	38.922	1550	1554	[27]
33	3,7-Dimethyl-1,5,7-octatrien-3-ol (Hotrienol)	96	41.425	1610	1603	[29]
43	3-Cyclohexene-1-methanol,α,α,4-trimethyl-(α-Terpineol)	87	44.322	1697	1694	[29]
45	1,1,6-Trimethyl-1,2-dihydronaphthalene (TDN)	85	45.774	1747	1737	[28]
50	(*E*)-1-(2,6,6-trimethyl-1,3-cyclohexadien-1-yl)-2-buten-1-one (β-Damascenone)	96	47.843	1823	1821	[29]
53	(*E*)-6,10-dimethyl-5,9-Undecadien-2-one (Geranylacetone)	97	48.627	1855	1855	[29]
64	(*E*,*E*)-3,7,11-trimethyl-2,6,10-Dodecatrien-1-ol ((*E*,*E*)-Farnesol)	91	58.976	2347	2366	[29]

RT—retention time; RTPRI exp.—retention index experimentally determined; RTPRI lit.—retention index reported in the literature for a CP-Wax columns or equivalent stationary phase.

**Table 2:** Classification accuracy of SVM method in the test set and in the subsets of Rondo and Zweigelt wines (%).

	Accuracy		
Compound	Test Set	Rondo	Zweigelt
Acids	93.33	87.50	100
Alcohols	100	100	100
Esters	86.67	71.43	100
Terpenes	93.33	100	83.33
Others	86.67	87.50	85.71
All	100	100	100

**Table 3:** Classification accuracy of kNN method in the test set and in the subsets of Rondo and Zweigelt wines (%).

	Accuracy		
Compound	Test Set	Rondo	Zweigelt
Acids ^1^	86.67	87.50	85.71
Alcohols ^1^	100	100	100
Esters	93.33	100	88.89
Terpenes ^1^	93.33	90	100
Others	80	72.73	100
All	100	100	100

1—for these compounds k = 1 for KNN method; for the remaining compounds k = 3.

**Table 4:** Subsets formed from selected compounds.

		Accuracy (%)	
Notations	Compounds	SVM	kNN
O1	octen-3-ol	73.33	73.33
O2	3-ethyl-4-methylpentan-1-ol	100	100
O3	butane-2,3-diol	86.67	60.00
O4	2-phenylethyl acetate	93.33	53.33
O5	3,7-dimethyl-1,5,7-octatrien-3-ol (hotrienol)	100	100
O6	3-(methylsulfanyl)propan-1-ol	86.67	80.00
T1	octen-3-ol; butane-2,3-diol	80.00	73.33
T2	octen-3-ol; 2-phenylethyl acetate	73.33	73.33
T3	octen-3-ol; 3-(methylsulfanyl)propan-1-ol	80.00	73.33
T4	butane-2,3-diol; 2-phenylethyl acetate	86.67	80.00
T5	butane-2,3-diol; 3-(methylsulfanyl)propan-1-ol	93.33	93.33
T6	2-phenylethyl acetate; 3-(methylsulfanyl)propan-1-ol	100	86.67
TR1	octen-3-ol; butane-2,3-diol; 2-phenylethyl acetate	80.00	86.67
TR2	octen-3-ol; butane-2,3-diol; 3-(methylsulfanyl)propan-1-ol	73.33	80.00
TR3	octen-3-ol; 2-phenylethyl acetate; 3-(methylsulfanyl)propan-1-ol	80.00	86.67
TR4	butane-2,3-diol; 2-phenylethyl acetate; 3-(methylsulfanyl)propan-1-ol	93.33	93.33
F	octen-3-ol; butane-2,3-diol; 2-phenylethyl acetate; 3-(methylsulfanyl)propan-1-ol	80.00	86.67

O1–O6—denote the one-element groups; T1–T6—groups including two elements; TR1–TR4—groups including three elements; F—the four-element group.

**Table 5:** Description of wine samples.

Wine Code	Grape Variety	Yeast	Lactic Acid Bacteria
Z1	Zweigelt	SafŒno™ SC 22	-
Z1 LAB	Zweigelt	SafŒno™ SC 22	Viniflora Oenos
Z2	Zweigelt	SafŒno™ HD S62	-
Z2 LAB	Zweigelt	SafŒno™ HD S62	Viniflora Oenos
Z3	Zweigelt	Essentiale Grand Cru	-
Z3 LAB	Zweigelt	Essentiale Grand Cru	Viniflora Oenos
Z4	Zweigelt	Siha Active Yeast 8	-
Z4 LAB	Zweigelt	Siha Active Yeast 8	Viniflora Oenos
Z5	Zweigelt	Siha Rubino Cru	-
Z5 LAB	Zweigelt	Siha Rubino Cru	Viniflora Oenos
R1	Rondo	SafŒno™ SC 22	-
R1 LAB	Rondo	SafŒno™ SC 22	Viniflora Oenos
R2	Rondo	SafŒno™ HD S62	-
R2 LAB	Rondo	SafŒno™ HD S62	Viniflora Oenos
R3	Rondo	Essentiale Grand Cru	-
R3 LAB	Rondo	Essentiale Grand Cru	Viniflora Oenos
R4	Rondo	Siha Active Yeast 8	-
R4 LAB	Rondo	Siha Active Yeast 8	Viniflora Oenos
R5	Rondo	Siha Rubino Cru	-
R5 LAB	Rondo	Siha Rubino Cru	Viniflora Oenos

## Data Availability

Not applicable.

## Supplementary Materials

The following supporting information can be downloaded at: https://www.mdpi.com/article/10.3390/molecules28041961/s1, Table S1: Relative concentrations of volatile compounds in Zweigelt wines (μg/L); Table S2: Relative concentrations of volatile compounds in Rondo wines (μg/L); Figure S1: GC/MS chromatogram of volatile compounds of Zweigelt wine (see Table 1 for compound names); Figure S2: GC/MS chromatogram of volatile compounds of Rondo wine (see Table 1 for compound names); Table S3: *p*-values obtained by the Mann-Whitney test when comparing Zweigelt and Rondo wines; Figure S3: Projection of variables on the PCA plane defined by the first two principal components (numbers 1–67 stand for volatile compounds; see Table 1 for their names).
