# Supplementary material for: Authentication of Polish Red Wines Produced from Zweigelt and Rondo Grape Varieties Based on Volatile Compounds Analysis in Combination with Machine Learning Algorithms: Hotrienol as a Marker of the Zweigelt Variety
Source: Molecules. 2023 Feb 18;28(4):1961. doi: 10.3390/molecules28041961 (PMC9967794; doi:10.3390/molecules28041961)
Supplement: Supplementary file 1 [file molecules-28-01961-s001.zip › molecules-2191332-supplementary.pdf]

Table S1. Relative concentrations of volatile compounds in Zweigelt wines (μg/L).

Compound	Wine											
	Z1	Z2	Z3	Z4	Z5	Mean	Z1 LAB	Z2 LAB	Z3 LAB	Z4 LAB	Z5 LAB	Mean	Total mean
Acids													
Acetic acid	21.66	23.53	44.43	28.36	37.28	31.05 ± 9.51	57.51	47.54	53.76	55.66	63.07	55.51 ± 7.59	43.28 ◎ 15.04
Propanoic acid	0.27	0.12	0.00	0.20	0.00	0.12 ± 0.18	0.00	0.09	0.22	0.00	0.23	0.11 ± 0.16	0.11 ± 0.16
2-Methylpropanoic acid	10.90	8.33	6.45	6.78	5.57	7.61 ± 6.41	11.63	5.03	3.21	7.30	8.81	7.20 ± 5.63	7.40 ± 5.93
Hexanoic acid	73.44	63.83	50.29	77.44	99.79	72.96 ± 19.94	56.86	54.35	63.65	91.35	70.12	67.27 ± 18.04	70.11 ± 18.91
Octanoic Acid	139.44	116.70	93.58	140.07	192.64	136.48 ± 40.17	103.67	105.98	116.77	170.83	120.82	123.61 ± 34.05	130.05 ± 37.17
Nonanoic acid	0.38	1.03	0.45	0.97	0.81	0.73 ± 0.41	0.69	0.52	0.67	0.43	0.60	0.58 ± 0.20	0.66 ± 0.33
Decanoic acid	11.22	9.80	7.28	18.34	22.41	13.81 ± 6.49	9.01	10.72	9.09	16.09	9.95	10.97 ± 3.69	12.39 ± 5.38
Benzoic acid	0.00	0.00	0.07	0.40	0.06	0.11 ± 0.16	0.12	0.04	0.55	0.06	0.03	0.16 ± 0.21	0.13 ± 0.19
Dodecanoic acid	0.00	0.08	0.08	0.28	0.16	0.12 ± 0.19	0.11	0.20	0.30	0.07	0.30	0.20 ± 0.18	0.16 ± 0.18
Subtotal	257.31	223.42	202.63	272.84	358.72		239.60	224.47	248.22	341.79	273.93		
Proportions (%)	6.69	7.43	10.29	10.35	8.42		8.47	9.41	9.52	12.73	9.07		
Alcohols													
2-Methylpropan-1-ol	183.07	17.55	104.37	160.00	157.55	124.51 ± 80.44	116.56	70.21	89.02	67.02	179.81	104.52 ± 54.41	114.52 ± 68.24
Butan-1-ol	3.38	3.20	0.24	1.15	5.92	2.78 ± 2.53	1.05	1.11	1.24	1.80	1.44	1.33 ± 1.40	2.05 ± 2.14
3-Methylbutan-1-ol	2132.56	1824.56	858.97	1437.50	2541.89	1759.10 ± 679.25	1368.19	1181.60	1260.33	1187.00	1540.52	1307.53 ± 435.26	1533.31 ± 605.75
Pentan-1-ol	1.07	0.78	0.66	0.56	0.92	0.80 ± 0.20	0.63	0.58	0.61	0.61	0.62	0.61 ± 0.09	0.71 ± 0.18
4-Methylpentan-1-ol	3.82	3.12	2.54	1.78	3.65	2.98 ± 0.81	2.05	2.14	2.43	2.02	2.29	2.19 ± 0.34	2.58 ± 0.73
3-Methylpentan-1-ol	5.01	4.27	3.11	2.59	4.32	3.86 ± 0.96	2.71	2.90	2.99	2.74	2.67	2.80 ± 0.34	3.33 ± 0.89
Hexan-1-ol	113.11	85.37	69.49	69.57	95.76	86.66 ± 18.47	63.47	61.10	65.59	83.37	62.74	67.25 ± 13.64	76.96 ± 18.76
E)-3-Hexen-1-ol	0.00	0.00	0.34	0.00	0.00	0.07 ± 0.18	0.00	0.00	0.17	0.13	0.00	0.06 ± 0.16	0.06 ± 0.17
3-Ethoxypropan-1-ol	0.18	0.54	0.53	0.51	0.54	0.46 ± 0.15	0.19	0.55	0.59	0.49	0.46	0.46 ± 0.16	0.46 ± 0.15
(Z)-3-Hexen-1-ol	0.00	0.07	0.14	0.12	0.24	0.11 ± 0.11	0.05	0.15	0.00	0.15	0.17	0.10 ± 0.09	0.11 ± 0.10
Octen-3-ol	0.00	0.00	0.00	0.00	1.17	0.23 ± 0.49	0.00	0.23	0.00	0.55	0.23	0.20 ± 0.47	0.22 ± 0.47
Heptan-1-ol	2.36	1.82	2.09	1.74	2.33	2.07 ± 0.34	1.42	1.72	2.20	2.91	1.80	2.01 ± 0.74	2.04 ± 0.56
2-Ethylhexan-1-ol	10.29	8.89	9.51	5.83	9.45	8.79 ± 2.28	4.21	5.83	9.72	8.10	5.49	6.67 ± 2.94	7.73 ± 2.80
3-Ethyl-4-methylpentan-1-ol	1.83	1.55	1.04	0.99	1.63	1.41 ± 0.38	0.80	0.99	1.06	1.38	0.88	1.02 ± 0.33	1.21 ± 0.40
Butane-2,3-diol	41.22	54.31	11.55	28.66	67.70	40.69 ± 28.38	25.80	39.27	57.67	30.94	34.60	37.66 ± 23.75	39.17 ± 25.76

Table S1. Cont.

Compound	Wine												
	Z1	Z2	Z3	Z4	Z5	Mean	Z1 LAB	Z2 LAB	Z3 LAB	Z4 LAB	Z5 LAB	Mean	Total mean
Octan-1-ol	22.29	8.90	11.86	8.26	21.36	14.53 ± 6.91	12.32	7.60	12.42	13.11	14.38	11.96 ± 4.39	13.25 ± 5.84
Propane-1,2-diol	0.00	0.18	0.12	0.67	0.65	0.32 ± 0.42	0.20	0.36	0.46	0.42	0.38	0.36 ± 0.33	0.34 ± 0.37
2-(2-Ethoxyethoxy)-ethanol	2.67	2.47	1.50	1.79	3.07	2.30 ± 0.66	1.50	1.44	1.59	1.90	1.42	1.57 ± 0.38	1.94 ± 0.65
Nonan-1-ol	4.75	2.94	2.50	2.22	4.28	3.34 ± 1.50	2.37	2.04	2.54	2.83	2.36	2.43 ± 1.07	2.88 ± 1.36
Decan-1-ol	3.56	1.76	2.30	1.43	4.31	2.67 ± 1.70	2.28	1.41	2.46	2.20	2.21	2.11 ± 1.09	2.39 ± 1.43
Phenylmethanol	2.69	1.13	0.22	0.99	0.24	1.05 ± 0.96	0.75	0.25	0.16	0.25	0.14	0.31 ± 0.24	0.68 ± 0.78
2-Phenylethanol	480.42	328.89	240.62	369.96	484.11	380.80 ± 110.00	419.99	294.15	326.85	405.73	354.08	360.16 ± 80.15	370.48 ± 95.14
Dodecan-1-ol	2.52	2.47	1.32	1.69	1.65	1.93 ± 0.95	0.62	0.88	1.24	1.39	0.75	0.98 ± 0.58	1.45 ± 0.91
Hexadecan-1-ol	0.45	0.19	0.30	0.53	0.00	0.29 ± 0.21	0.00	0.19	0.00	0.39	0.00	0.12 ± 0.18	0.21 ± 0.21
Subtotal	3017.25	2354.96	1325.32	2098.54	3412.74		2027.16	1676.70	1841.34	1817.43	2209.44		
Proportions (%)	78.39	78.36	67.28	79.63	80.10		71.70	70.26	70.63	67.70	73.16		
Aldehydes													
Benzaldehyde	0.00	0.00	13.81	0.11	14.58	5.70 ± 7.24	6.80	6.70	3.69	7.59	10.44	7.04 ± 2.42	6.37 ± 5.35
4-Methylbenzaldehyde	0.80	0.60	0.48	0.77	0.68	0.67 ± 0.14	0.30	0.43	0.31	0.69	0.45	0.44 ± 0.17	0.55 ± 0.19
Subtotal	0.80	0.60	14.29	0.88	15.26		7.10	7.13	4.00	8.28	10.89		
Proportions (%)	0.02	0.02	0.73	0.03	0.36		0.25	0.30	0.15	0.31	0.36		
Esters													
Ethyl 3-methylbutanoate	2.00	0.45	0.64	0.83	1.51	1.09 ± 0.68	0.96	0.72	0.60	0.42	1.04	0.75 ± 0.28	0.92 ± 0.54
Ethyl 2-hydroxypropanoate	87.24	107.41	202.62	41.48	85.73	104.90 ± 58.13	295.83	286.35	252.23	247.97	286.39	273.75 ± 47.80	189.32 ± 100.54
Methyl octanoate	0.61	0.41	0.14	0.50	0.09	0.35 ± 0.37	0.14	0.06	0.05	0.00	0.07	0.07 ± 0.13	0.21 ± 0.30
Ethyl octanoate	139.24	96.59	54.54	79.72	110.60	96.14 ± 42.11	79.58	33.64	65.23	120.55	66.30	73.06 ± 54.06	84.60 ± 49.04
2-Methylpropyl 2-hydroxypropanoate	0.44	0.34	0.78	0.22	0.50	0.45 ± 0.21	1.14	0.70	0.90	1.21	1.27	1.04 ± 0.28	0.75 ± 0.39
Ethyl nonanoate	0.58	0.46	0.23	0.31	0.33	0.38 ± 0.18	0.34	0.14	0.19	0.32	0.10	0.22 ± 0.18	0.30 ± 0.19
3-Methylbutyl 2-hydroxypropanoate	3.02	3.29	5.07	1.18	3.60	3.23 ± 1.36	7.64	7.79	6.90	8.89	9.52	8.15 ± 1.70	5.69 ± 2.92
Ethyl decanoate	13.79	10.23	10.32	12.22	17.84	12.88 ± 9.87	10.90	6.85	6.76	16.11	7.85	9.69 ± 10.03	11.29 ± 9.91
Ethyl benzoate	0.18	0.35	0.07	0.12	0.22	0.19 ± 0.13	0.09	0.09	0.28	0.15	0.05	0.13 ± 0.12	0.16 ± 0.13
Diethyl butanedioate	124.99	94.88	64.73	40.62	106.28	86.30 ± 34.92	69.41	58.89	69.00	47.44	64.25	61.80 ± 13.32	74.05 ± 28.80
Ethyl 9-decenoate	1.32	0.64	1.91	0.54	1.34	1.15 ± 0.92	0.61	0.33	1.52	0.53	0.64	0.73 ± 0.63	0.94 ± 0.81

Table S1. Cont.

Compound	Wine												
	Z1	Z2	Z3	Z4	Z5	Mean	Z1 LAB	Z2 LAB	Z3 LAB	Z4 LAB	Z5 LAB	Mean	Total mean
Methyl 2-hydroxy benzoate	0.17	0.12	0.16	0.09	0.14	0.14 ± 0.03	0.16	0.12	0.14	0.08	0.12	0.12 ± 0.04	0.13 ± 0.04
Ethyl phenylacetate	2.19	1.74	0.90	1.27	2.38	1.70 ± 0.60	1.10	1.14	0.94	1.44	1.34	1.19 ± 0.28	1.45 ± 0.53
2-Phenylethyl acetate	0.00	0.00	0.00	0.62	0.00	0.12 ± 0.48	4.05	2.97	0.00	0.00	3.58	2.12 ± 1.85	1.12 ± 1.67
Ethyl dodecanoate	0.00	0.33	0.00	0.23	0.52	0.22 ± 0.48	0.42	0.05	0.00	0.03	0.13	0.12 ± 0.19	0.17 ± 0.36
Ethyl 3-phenylpropanoate	0.46	0.37	0.74	0.65	2.45	0.93 ± 0.82	0.30	0.30	0.87	0.91	1.55	0.79 ± 0.52	0.86 ± 0.68
Diethyl-2-hydroxybutanedioate	0.35	0.22	0.00	0.28	0.42	0.25 ± 0.15	0.00	0.00	0.03	0.03	0.02	0.01 ± 0.03	0.13 ± 0.16
Methyl hexadecanoate	0.10	0.23	0.07	0.39	0.00	0.16 ± 0.23	0.12	0.00	0.06	0.00	0.00	0.04 ± 0.08	0.10 ± 0.18
Ethyl hexadecanoate	0.20	0.22	0.13	0.04	0.38	0.20 ± 0.13	0.10	0.15	0.06	0.12	0.14	0.11 ± 0.08	0.16 ± 0.12
Subtotal	376.88	318.28	343.05	181.31	334.33		472.89	400.29	405.76	446.2	444.36		
Proportions (%)	9.79	10.59	17.42	6.88	7.85		16.73	16.77	15.56	16.62	14.71		
Furan compounds													
Ethyl 2-furoate	0.71	0.55	0.37	0.36	0.64	0.53 ± 0.16	0.32	0.29	0.32	0.33	0.32	0.32 ± 0.05	0.42 ± 0.16
Dihydrofuran-2(3H)-one	0.00	0.00	0.00	0.59	0.00	0.12 ± 0.46	0.95	0.57	0.88	1.28	0.85	0.91 ± 1.18	0.51 ± 0.97
Subtotal	0.71	0.55	0.37	0.95	0.64		1.27	0.86	1.2	1.61	1.17		
Proportions (%)	0.02	0.02	0.02	0.04	0.02		0.04	0.04	0.05	0.06	0.04		
Ketones													
4-Methyl-3-penten-2-one	139.07	100.30	78.26	75.05	130.17	104.57 ± 56.43	74.99	72.83	99.53	61.88	75.33	76.91 ± 33.24	90.74 ± 47.63
3-Hydroxybutan-2-one	0.25	0.41	0.37	0.27	0.40	0.34 ± 0.13	0.55	0.24	0.60	0.37	0.32	0.42 ± 0.16	0.38 ± 0.15
Subtotal	139.32	100.71	78.63	75.32	130.57		75.54	73.07	100.13	62.25	75.65		
Proportions (%)	3.62	3.35	3.99	2.86	3.06		2.67	3.06	3.84	2.32	2.51		
Sulphur compounds													
3-(Methylsulfanyl)propan-1-ol	1.46	0.67	0.85	1.23	1.33	1.11 ± 0.33	1.63	0.66	1.10	1.27	0.93	1.12 ± 0.38	1.11 ± 0.35
Subtotal	1.46	0.67	0.85	1.23	1.33		1.63	0.66	1.10	1.27	0.93		
Proportions (%)	0.04	0.02	0.04	0.05	0.03		0.06	0.03	0.04	0.05	0.03		
Terpenes													
(*Z*)-Linalool oxide	0.33	0.24	0.17	0.18	0.24	0.23 ± 0.19	0.06	0.20	0.26	0.19	0.18	0.18 ± 0.14	0.20 ± 0.16

Table S1. Cont.

Compound	Wine											
	Z1	Z2	Z3	Z4	Z5	Mean	Z1 LAB	Z2 LAB	Z3 LAB	Z4 LAB	Z5 LAB	Mean	Total mean
3,7-Dimethyl-1,6-octadien-3-ol (β-Linalol)	0.13	0.14	0.20	0.11	0.18	0.15 ± 0.23	0.14	0.13	0.09	0.24	0.12	0.14 ± 0.22	0.15 ± 0.22
3,7-Dimethyl-1,5,7-octatrien-3-ol (Hotrienol)	0.67	0.54	0.59	0.47	0.82	0.62 ± 0.36	0.52	0.50	0.53	0.79	0.58	0.58 ± 0.30	0.60 ± 0.33
3-Cyclohexene-1-methanol, alpha,alpha,4-trimethyl- (α-Terpineol)	0.61	0.49	0.56	0.31	0.63	0.52 ± 0.28	0.39	0.34	0.47	0.55	0.28	0.41 ± 0.32	0.46 ± 0.30
1,1,6-Trimethyl-1,2-dihydronaphthalene (TDN)	1.45	1.91	0.98	1.18	2.25	1.55 ± 1.07	0.72	0.73	2.04	0.91	1.07	1.09 ± 0.77	1.32 ± 0.94
(*E*)-1-(2,6,6-trimethyl-1,3-cyclohexadien-1-yl)-2-buten-1-one (β-Damascenone)	2.70	2.23	1.44	1.61	2.96	2.19 ± 0.72	0.00	0.95	1.71	2.13	0.95	1.15 ± 0.94	1.67 ± 0.98
(*E*)-6,10-dimethyl-5,9-Undecadien-2-one (Geranylacetone)	47.31	0.00	0.00	0.00	0.00	9.46 ± 26.17	0.00	0.00	0.00	0.00	0.00	0.00 ± 0.00	4.73 ± 18.81
(*E*,*E*)-3,7,11-trimethyl-2,6,10-Dodecatrien-1-ol ((*E*,*E*)-Farnesol)	1.85	0.58	0.73	0.34	0.12	0.72 ± 0.69	0.16	0.43	0.31	0.74	0.29	0.38 ± 0.29	0.55 ± 0.55
Subtotal	55.05	6.13	4.67	4.20	7.20		1.99	3.28	5.41	5.55	3.47		
Proportions (%)	1.43	0.20	0.24	0.16	0.17		0.07	0.14	0.21	0.21	0.11		
Total	3848.78	3005.32	1969.81	2635.27	4260.79		2827.18	2386.46	2607.16	2684.38	3019.84		

Z1-Z5 - Zweigelt wines, in which AF was induced using various yeast strains, and the wines were left to undergo spontaneous MLF; Z1 LAB-Z5 LAB – Zweigelt wines, in which AF was induced using various yeast strains (but the same strains as in Z1-Z5 wines), and MLF was carried out by inoculation with lactic acid bacteria

Table S2. Relative concentrations of volatile compounds in Rondo wines (μg/L).

Compound	Wine												
	R1	R2	R3	R4	R5	Mean	R1 LAB	R2 LAB	R3 LAB	R4 LAB	R5 LAB	Mean	Total mean
Acids													
Acetic acid	25.07	36.03	32.62	34.92	54.87	36.71 ± 12.01	79.84	102.56	101.98	106.36	94.52	97.05 ± 16.15	66.88 ± 33.72
Propanoic acid	0.12	0.00	0.30	0.19	0.00	0.12 ± 0.19	0.33	0.00	0.00	0.17	0.00	0.10 ± 0.15	0.11 ± 0.17
2-Methylpropanoic acid	0.00	0.00	10.00	9.01	8.90	5.58 ± 8.71	9.48	8.06	4.68	4.68	14.22	8.22 ± 8.43	6.90 ± 8.53
Hexanoic acid	33.57	29.73	61.68	48.43	53.48	45.38 ± 14.03	39.40	28.29	37.58	37.88	39.02	36.43 ± 6.58	40.91 ± 11.69
Octanoic acid	60.92	58.66	126.06	107.56	112.88	93.21 ± 35.66	77.08	56.15	73.04	78.49	74.87	71.93 ± 14.48	82.57 ± 28.85
Nonanoic acid	0.75	0.43	1.03	1.02	1.45	0.94 ± 0.43	1.19	0.74	0.77	0.65	0.77	0.82 ± 0.36	0.88 ± 0.40
Decanoic acid	9.11	11.86	16.75	17.55	17.24	14.50 ± 5.07	11.47	7.57	7.97	8.94	6.64	8.52 ± 2.75	11.51 ± 5.03
Benzoic acid	0.15	0.16	0.16	0.14	0.19	0.16 ± 0.05	0.15	0.14	0.23	0.06	0.09	0.13 ± 0.08	0.15 ± 0.07
Dodecanoic acid	0.33	0.14	0.61	0.47	1.03	0.51 ± 0.42	0.23	0.00	0.00	0.28	0.00	0.10 ± 0.24	0.31 ± 0.39
Subtotal	130.02	137.01	249.21	219.29	250.04		219.17	203.51	226.25	237.51	230.13		
Proportions (%)	4.29	6.87	6.62	9.24	6.50		6.79	7.19	11.39	6.34	7.19		
Alcohols													
2-Methylpropan-1-ol	116.86	127.81	302.81	112.29	311.17	194.19 ± 138.66	204.20	62.30	96.60	207.85	245.60	163.31 ± 140.27	178.75 ± 137.94
Butan-1-ol	2.88	1.35	3.60	0.49	3.67	2.40 ± 2.00	4.55	0.45	0.00	1.30	0.49	1.36 ± 1.95	1.88 ± 2.01
3-Methylbutan-1-ol	1873.26	1226.39	2228.80	1553.77	2306.31	1837.71 ± 573.99	1658.73	1522.54	784.94	2052.41	1706.13	1544.95 ± 584.30	1691.33 ± 588.25
Pentan-1-ol	0.36	0.45	0.66	0.26	0.59	0.46 ± 0.22	0.30	0.44	0.22	0.55	0.52	0.41 ± 0.20	0.43 ± 0.21
4-Methylpentan-1-ol	2.25	1.65	2.29	0.68	1.74	1.72 ± 0.71	2.22	1.25	1.08	1.26	1.22	1.41 ± 0.50	1.56 ± 0.62
3-Methylpentan-1-ol	4.02	2.79	4.03	1.98	2.02	2.96 ± 1.07	3.55	2.01	1.87	2.87	1.53	2.37 ± 0.90	2.67 ± 1.02
Hexan-1-ol	54.40	46.72	73.94	47.66	62.30	57.00 ± 15.28	60.22	46.47	40.47	72.23	51.45	54.17 ± 15.52	55.59 ± 15.20
E)-3-Hexen-1-ol	0.18	0.00	0.00	0.00	0.00	0.04 ± 0.14	0.00	0.15	0.26	0.00	0.00	0.08 ± 0.17	0.06 ± 0.15
3-Ethoxypropan-1-ol	0.17	0.50	0.58	0.22	0.36	0.37 ± 0.17	0.16	0.40	0.49	0.29	0.30	0.33 ± 0.12	0.35 ± 0.15
(*Z*)-3-Hexen-1-ol	0.09	0.09	0.30	0.00	0.13	0.12 ± 0.12	0.23	0.15	0.08	0.34	0.17	0.19 ± 0.13	0.16 ± 0.13
Octen-3-ol	1.21	1.25	1.72	1.17	1.71	1.41 ± 0.47	1.51	1.01	0.82	1.67	0.47	1.09 ± 0.68	1.25 ± 0.60
Heptan-1-ol	1.24	1.12	2.22	1.17	1.61	1.47 ± 0.56	1.63	1.09	1.21	1.99	1.14	1.41 ± 0.53	1.44 ± 0.54
2-Ethylhexan-1-ol	10.03	6.44	10.68	3.32	11.39	8.37 ± 4.11	10.57	4.34	2.30	3.85	6.64	5.54 ± 3.91	6.96 ± 4.20
3-Ethyl-4-methylpentan-1-ol	0.23	0.24	0.16	0.21	0.08	0.18 ± 0.10	0.24	0.16	0.08	0.26	0.11	0.17 ± 0.11	0.18 ± 0.11
Butane-2,3-diol	41.22	54.31	11.55	28.66	67.70	40.69 ± 28.38	25.80	39.27	57.67	30.94	34.60	37.66 ± 23.75	39.17 ± 25.76
Octan-1-ol	6.80	4.06	8.04	4.83	7.75	6.30 ± 2.53	10.29	4.96	6.11	9.71	7.40	7.70 ± 3.63	7.00 ± 3.16
Propane-1,2-diol	1.67	1.22	0.96	0.57	1.97	1.28 ± 0.99	0.98	0.42	3.85	0.86	0.72	1.36 ± 2.04	1.32 ± 1.58

Table S2. Cont.

Compound	Wine												
	R1	R2	R3	R4	R5	Mean	R1 LAB	R2 LAB	R3 LAB	R4 LAB	R5 LAB	Mean	Total mean
2-(2-Ethoxyethoxy)- ethanol	4.85	9.59	4.02	1.72	2.42	4.52 ± 3.85	2.24	1.84	1.40	1.93	1.42	1.77 ± 0.51	3.14 ± 3.04
Nonan-1-ol	4.23	4.34	6.83	4.63	5.74	5.16 ± 2.08	4.52	2.77	2.94	5.08	2.88	3.64 ± 2.13	4.40 ± 2.21
Decan-1-ol	1.65	1.29	2.54	1.38	1.90	1.75 ± 0.93	2.25	1.44	1.57	2.28	1.37	1.78 ± 1.05	1.77 ± 0.97
Phenylmethanol	0.45	0.19	0.11	0.08	0.19	0.20 ± 0.15	0.10	0.12	0.08	0.07	0.18	0.11 ± 0.08	0.16 ± 0.12
2-Phenylethanol	533.10	107.00	509.82	168.80	480.53	359.85 ± 218.36	399.50	390.77	327.89	436.47	373.40	385.61 ± 102.61	372.73 ± 168.15
Dodecan-1-ol	0.86	1.80	1.53	0.95	1.85	1.40 ± 0.69	1.12	2.90	1.07	1.81	0.72	1.53 ± 1.29	1.46 ± 1.02
Hexadecan-1-ol	0.35	0.34	0.46	0.15	0.86	0.43 ± 0.32	0.37	0.07	0.15	0.38	0.00	0.19 ± 0.19	0.31 ± 0.28
Subtotal	2662.36	1600.94	3177.65	1934.99	3273.99		2395.28	2087.32	1333.15	2836.4	2438.46		
Proportions (%)	87.78	80.24	84.42	81.54	85.11		74.17	73.72	67.12	75.73	76.20		
Aldehydes													
Benzaldehyde	1.32	3.02	4.37	2.05	4.32	3.02 ± 1.45	2.74	3.99	4.07	3.64	4.82	3.85 ± 0.94	3.43 ± 1.28
4-Methylbenzaldehyde	2.91	0.66	2.44	1.57	1.19	1.75 ± 0.88	0.90	0.52	0.71	0.53	0.45	0.62 ± 0.22	1.19 ± 0.85
Subtotal	4.23	3.68	6.81	3.62	5.51		3.64	4.51	4.78	4.17	5.27		
Proportions (%)	0.14	0.18	0.18	0.15	0.14		0.11	0.16	0.24	0.11	0.16		
Esters													
Ethyl 3-methylbutanoate	0.69	0.00	0.93	0.19	1.60	0.68 ± 0.69	0.79	0.58	0.12	0.18	0.85	0.51 ± 0.36	0.59 ± 0.55
Ethyl 2-hydroxypropanoate	35.08	45.94	55.89	49.15	39.84	45.18 ± 14.05	369.17	354.83	212.31	382.15	303.83	324.46 ± 99.21	184.82 ± 158.17
Methyl octanoate	0.10	0.19	0.05	0.14	0.00	0.10 ± 0.13	0.00	0.09	0.00	0.50	0.19	0.15 ± 0.30	0.12 ± 0.23
Ethyl octanoate	45.54	46.34	39.08	43.68	77.55	50.44 ± 25.15	48.96	44.10	70.27	62.62	64.28	58.05 ± 36.29	54.24 ± 30.92
2-Methylpropyl 2-hydroxypropanoate	0.21	0.15	0.37	0.29	0.30	0.27 ± 0.09	1.63	1.11	1.04	2.09	2.09	1.59 ± 0.63	0.93 ± 0.81
Ethyl nonanoate	0.55	0.40	0.73	0.38	0.62	0.54 ± 0.34	0.16	0.41	0.47	0.47	0.41	0.38 ± 0.25	0.46 ± 0.30
3-Methylbutyl 2-hydroxypropanoate	1.61	1.38	1.90	1.89	1.67	1.69 ± 0.26	13.36	8.77	7.71	12.89	10.69	10.68 ± 2.69	6.19 ± 4.94
Ethyl decanoate	9.45	7.30	16.97	8.27	15.72	11.54 ± 9.84	7.95	8.08	8.75	10.77	7.71	8.65 ± 6.95	10.10 ± 8.50
Ethyl benzoate	0.07	0.00	0.19	0.00	0.10	0.07 ± 0.09	0.03	0.00	0.00	0.00	0.03	0.01 ± 0.03	0.04 ± 0.08
Diethyl butanedioate	63.20	40.86	88.68	48.84	54.41	59.20 ± 18.04	61.37	41.18	50.47	43.06	42.43	47.70 ± 10.29	53.45 ± 15.57
Ethyl 9-decenoate	0.00	0.00	1.76	0.07	0.12	0.39 ± 0.94	0.12	0.00	1.01	0.00	0.07	0.24 ± 0.47	0.31 ± 0.73
Methyl 2-hydroxy benzoate	0.11	0.10	0.15	0.13	0.14	0.13 ± 0.03	0.31	0.26	0.24	0.34	0.32	0.30 ± 0.08	0.21 ± 0.11
Ethyl phenylacetate	1.68	1.39	1.63	1.59	2.05	1.67 ± 0.29	1.47	1.13	0.70	1.29	1.14	1.15 ± 0.35	1.41 ± 0.41

Table S2. Cont.

Compound	Wine												
	R1	R2	R3	R4	R5	Mean	R1 LAB	R2 LAB	R3 LAB	R4 LAB	R5 LAB	Mean	Total mean
2-Phenylethyl acetate	3.42	2.79	0.00	0.00	4.62	2.17 ± 2.91	8.72	6.54	4.14	9.16	7.69	7.25 ± 2.45	4.71 ± 3.70
Ethyl dodecanoate	0.35	0.00	0.54	0.00	0.26	0.23 ± 0.50	0.00	0.00	0.22	0.32	0.16	0.14 ± 0.26	0.19 ± 0.40
Ethyl 3-phenylpropanoate	0.34	0.29	1.33	0.91	1.42	0.86 ± 0.53	0.46	0.31	0.73	1.04	1.05	0.72 ± 0.36	0.79 ± 0.45
Diethyl-2-hydroxybutanedioate	0.74	0.40	0.63	0.55	0.87	0.64 ± 0.27	0.00	0.00	0.00	0.00	0.00	0.00 ± 0.00	0.32 ± 0.38
Methyl hexadecanoate	0.00	0.14	0.10	0.00	0.16	0.08 ± 0.12	0.00	0.07	0.00	0.15	0.09	0.06 ± 0.14	0.07 ± 0.13
Ethyl hexadecanoate	0.83	0.23	0.73	0.53	0.48	0.56 ± 0.29	0.18	0.05	0.05	0.22	0.12	0.12 ± 0.10	0.34 ± 0.31
Subtotal	163.97	147.90	211.66	156.61	201.93		514.68	467.51	358.23	527.25	443.15		
Proportions (%)	5.41	7.41	5.62	6.60	5.25		15.94	16.51	18.03	14.08	13.85		
Furan compounds													
Ethyl 2-furoate	0.52	0.42	0.63	0.43	0.61	0.52 ± 0.12	0.41	0.34	0.27	0.40	0.36	0.36 ± 0.08	0.44 ± 0.13
Dihydrofuran-2(3H)-one	1.04	1.43	2.18	0.00	0.00	0.93 ± 1.44	5.16	0.00	4.71	5.39	5.62	4.18 ± 3.02	2.55 ± 2.85
Subtotal	1.56	1.85	2.81	0.43	0.61		5.57	0.34	4.98	5.79	5.98		
Proportions (%)	0.05	0.09	0.07	0.02	0.02		0.17	0.01	0.25	0.15	0.19		
Ketones													
4-Methyl-3-penten-2-one	58.05	45.57	106.26	52.31	95.99	71.64 ± 38.22	80.16	57.28	55.50	87.73	73.57	70.85 ± 20.91	71.24 ± 30.28
3-Hydroxybutan-2-one	0.28	0.22	0.39	0.30	0.47	0.33 ± 0.11	0.19	0.23	0.40	0.36	0.18	0.27 ± 0.14	0.30 ± 0.13
Subtotal	58.33	45.79	106.65	52.61	96.46		80.35	57.51	55.90	88.09	73.75		
Proportions (%)	1.92	2.30	2.83	2.22	2.51		2.49	2.03	2.81	2.35	2.30		
Sulphur compounds													
3-(Methylsulfanyl)propan-1-ol	2.80	1.72	2.52	2.08	2.84	2.39 ± 0.54	3.11	1.51	1.85	1.57	1.99	2.01 ± 0.72	2.20 ± 0.65
Subtotal	2.80	1.72	2.52	2.08	2.84		3.11	1.51	1.85	1.57	1.99		
Proportions (%)	0.09	0.09	0.07	0.09	0.07		0.10	0.05	0.09	0.04	0.06		
Terpenes													
(*Z*)-Linalool oxide	0.00	33.28	0.00	0.00	0.00	6.66 ± 14.17	0.00	0.00	0.00	0.00	0.00	0.00 ± 0.00	3.33 ± 10.41
3,7-Dimethyl-1,6-octadien-3-ol (β-Linalol)	0.22	0.31	0.40	0.12	0.31	0.27 ± 0.36	0.32	0.32	0.00	0.00	0.13	0.15 ± 0.31	0.21 ± 0.34
3,7-Dimethyl-1,5,7-octatrien-3-ol (Hotrienol)	0.00	0.00	0.00	0.00	0.00	0.00 ± 0.00	0.00	0.00	0.00	0.00	0.00	0.00 ± 0.00	0.00 ± 0.00

Table S2. Cont.

Compound	Wine R1	R2	R3	R4	R5	Mean	R1 LAB	R2 LAB	R3 LAB	R4 LAB	R5 LAB	Mean	Total mean
3-Cyclohexene-1-methanol, alpha,alpha,4-trimethyl- (α-Terpineol)	0.46	0.54	0.65	0.12	0.39	0.43 ± 0.35	0.54	0.45	0.44	0.35	0.33	0.42 ± 0.19	0.43 ± 0.27
1,1,6-Trimethyl-1,2-dihydronaphthalene (TDN)	0.49	0.19	1.13	1.09	0.68	0.72 ± 0.67	0.36	0.33	0.73	0.63	0.59	0.53 ± 0.34	0.62 ± 0.53
(*E*)-1-(2,6,6-trimethyl-1,3-cyclohexadien-1-yl)-2-buten-1-one (β-Damascenone)	0.00	0.00	3.70	2.11	2.23	1.61 ± 1.84	1.05	0.67	0.00	0.00	0.00	0.34 ± 0.94	0.98 ± 1.57
(*E*)-6,10-dimethyl-5,9-Undecadien-2-one (Geranylacetone)	8.13	21.65	0.00	0.00	10.90	8.14 ± 14.83	4.78	6.95	0.00	42.47	0.00	10.84 ± 24.31	9.49 ± 19.83
(*E*,*E*)-3,7,11-trimethyl-2,6,10-Dodecatrien-1-ol ((*E*,*E*)-Farnesol)	0.27	0.24	0.72	0.06	0.73	0.40 ± 0.33	0.43	0.41	0.04	1.24	0.18	0.46 ± 0.44	0.43 ± 0.38
Subtotal	9.57	56.21	6.60	3.50	15.24		7.48	9.13	1.21	44.69	1.23		
Proportions (%)	0.32	2.82	0.18	0.15	0.40		0.23	0.32	0.06	1.19	0.04		
Total	3032.84	1995.10	3763.91	2373.13	3846.62		3229.28	2831.34	1986.35	3745.47	3199.96		

R1- R5 - Rondo wines, in which AF was induced using various yeast strains, and the wines were left to undergo spontaneous MLF; R1 LAB-R5 LAB - Rondo wines, in which AF was induced using various yeast strains (but the same strains as in R1-R5 wines), and MLF was carried out by inoculation with lactic acid bacteria

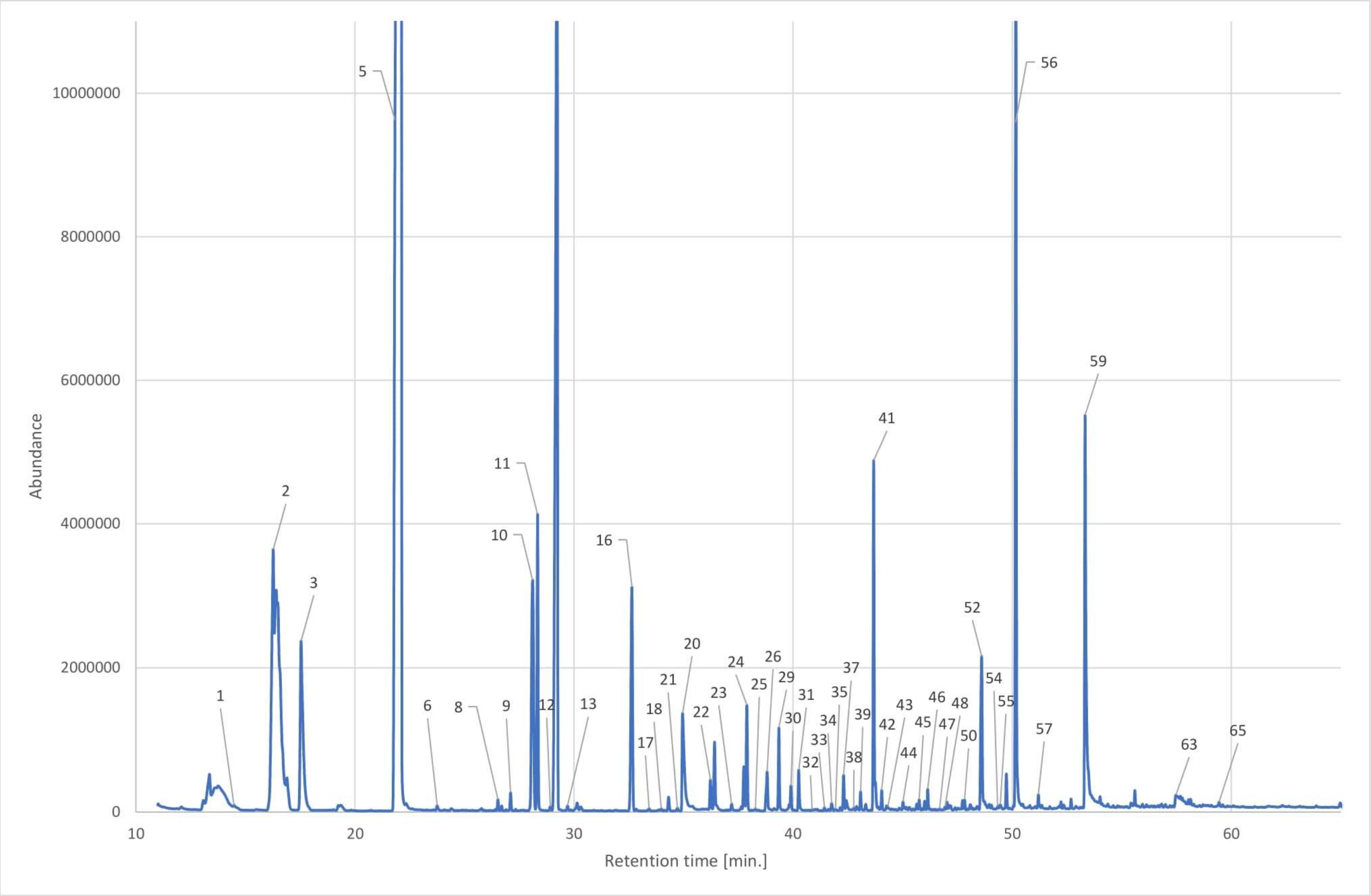

Figure S1. GC/MS chromatogram of volatile compounds of Zweigelt wine (see Table 1 for compound names).

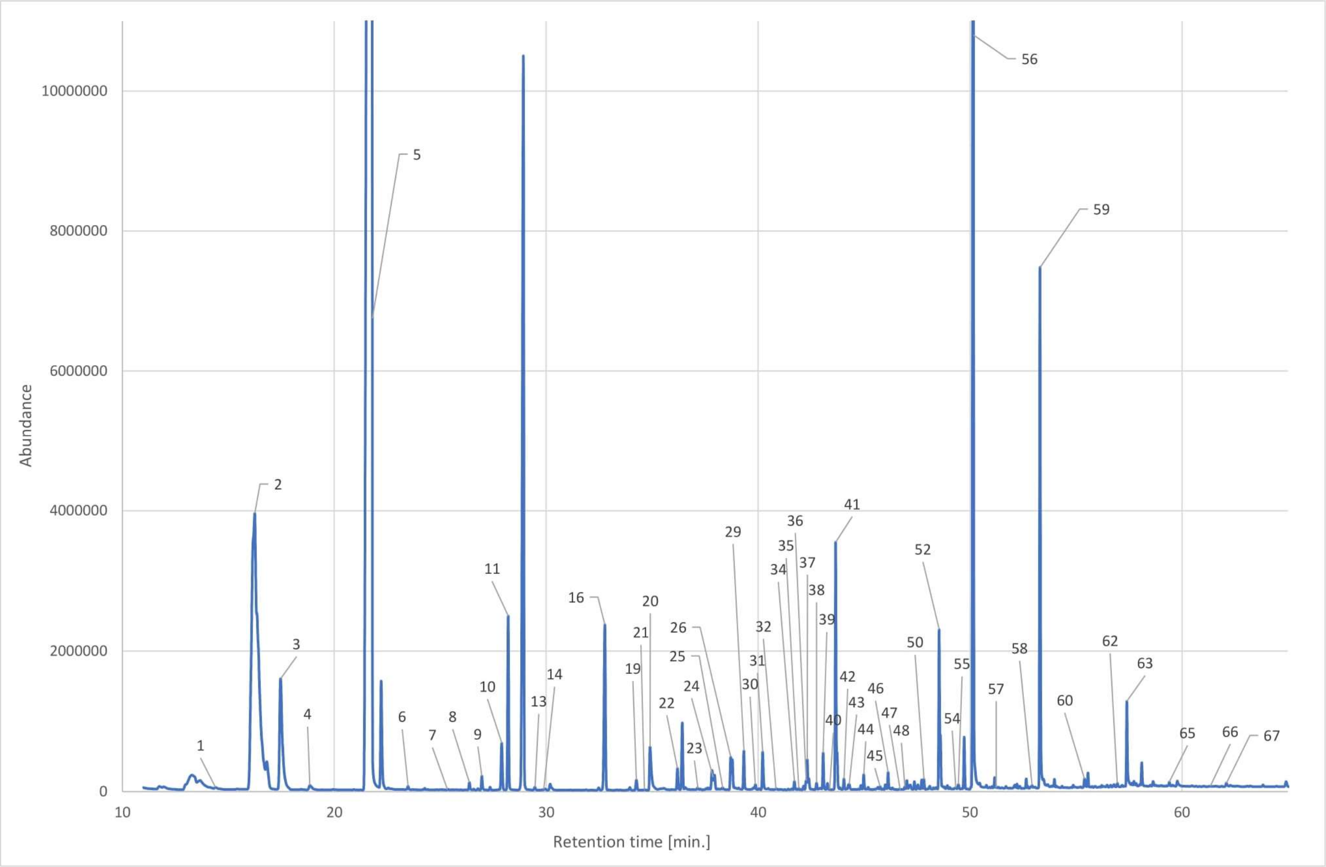

Figure S2. GC/MS chromatogram of volatile compounds of Rondo wine (see Table 1 for compound names).

Table S3. *p*-values obtained by the Mann-Whitney test when comparing Zweigelt and Rondo wines.

Compound	*p*-value	Compound	*p*-value	Compound	*p*-value
Acids		Butane-2,3-diol	0.00000005	Methyl 2-hydroxybenzoate	0.00463741
Acetic acid	0.01628537	Octan-1-ol	0.00000200	Ethyl phenylacetate	0.80727511
Propanoic acid	0.85319729	Propane-1,2-diol	0.00014849	2-Phenylethyl acetate	0.00006063
2-Methylpropanoic acid	0.90741272	2-(2-Ethoxyethoxy)- ethanol	0.10869076	Ethyl dodecanoate	1.00000000
Hexanoic acid	0.00000004	Nonan-1-ol	0.00205251	Ethyl 3-phenylpropanoate	0.97051607
Octanoic Acid	0.00000200	Decan-1-ol	0.07244652	Diethyl-2-hydroxybutanedioate	0.10025063
Nonanoic acid	0.02061967	Phenylmethanol	0.00001238	Methyl hexadecanoate	0.75895739
Decanoic acid	0.31118841	2-Phenylethanol	0.68432286	Ethyl hexadecanoate	0.01685781
Benzoic acid	0.01693276	Dodecan-1-ol	0.83025542	***Furan compounds***	
Dodecanoic acid	0.24539308	Hexadecan-1-ol	0.21656436	Ethyl 2-furoate	0.33285542
Alcohols		***Aldehydes***		Dihydrofuran-2(3H)-one	0.00204491
2-Methylpropan-1-ol	0.05010835	Benzaldehyde	0.03483315	***Ketones***	
Butan-1-ol	0.74600910	4-Methylbenzaldehyde	0.00044598	4-Methyl-3-penten-2-one	0.09923981
3-Methylbutan-1-ol	0.34026235	***Esters***		3-Hydroxybutan-2-one	0.03915697
Pentan-1-ol	0.00000267	Ethyl 3-methylbutanoate	0.02255067	***Sulphur compounds***	
4-Methylpentan-1-ol	0.00000268	Ethyl 2-hydroxypropanoate	0.75058409	3-(Methylsulfanyl)propan-1-ol	0.00000000
3-Methylpentan-1-ol	0.00367114	Methyl octanoate	0.35846808	***Terpenes***	
Hexan-1-ol	0.00000546	Ethyl octanoate	0.00303417	(*Z*)-Linalool oxide	0.00014867
(*E*)-3-Hexen-1-ol	0.97005940	2-Methylpropyl 2-hydroxypropanoate	0.94695598	3,7-Dimethyl-1,6-octadien-3-ol (*β*-Linalol)	0.81956394
3-Ethoxypropan-1-ol	0.00262448	Ethyl nonanoate	0.01694934	3,7-Dimethyl-1,5,7-octatrien-3-ol Hotrienol	0.00000000
(*Z*)-3-Hexen-1-ol	0.06667943	3-Methylbutyl 2-hydroxypropanoate	0.92344219	3-Cyclohexene-1-methanol, alpha,alpha,4-trimethyl- (*α*-Terpineol)	0.55802517
Octen-3-ol	0.00000004	Ethyl decanoate	0.87663500	1,1,6-Trimethyl-1,2-dihydronaphthalene (TDN)	0.00105767
Heptan-1-ol	0.00002133	Ethyl benzoate	0.00013965	(*E*)-1-(2,6,6-trimethyl-1,3-cyclohexadien-1-yl)-2-buten-1-one *β*-Damascenone	0.02244127
2-Ethylhexan-1-ol	0.22257380	Diethyl butanedioate	0.00195285	(*E*)-6,10-dimethyl-5,9-Undecadien-2-one (Geranylacetone)	0.06024460
3-Ethyl-4-methylpentan-1-ol	0.00000000	Ethyl 9-decenoate	0.00000160	(*E*,*E*)-3,7,11-trimethyl-2,6,10-Dodecatrien-1-ol ((*E*,*E*)-Farnesol)	0.58796458

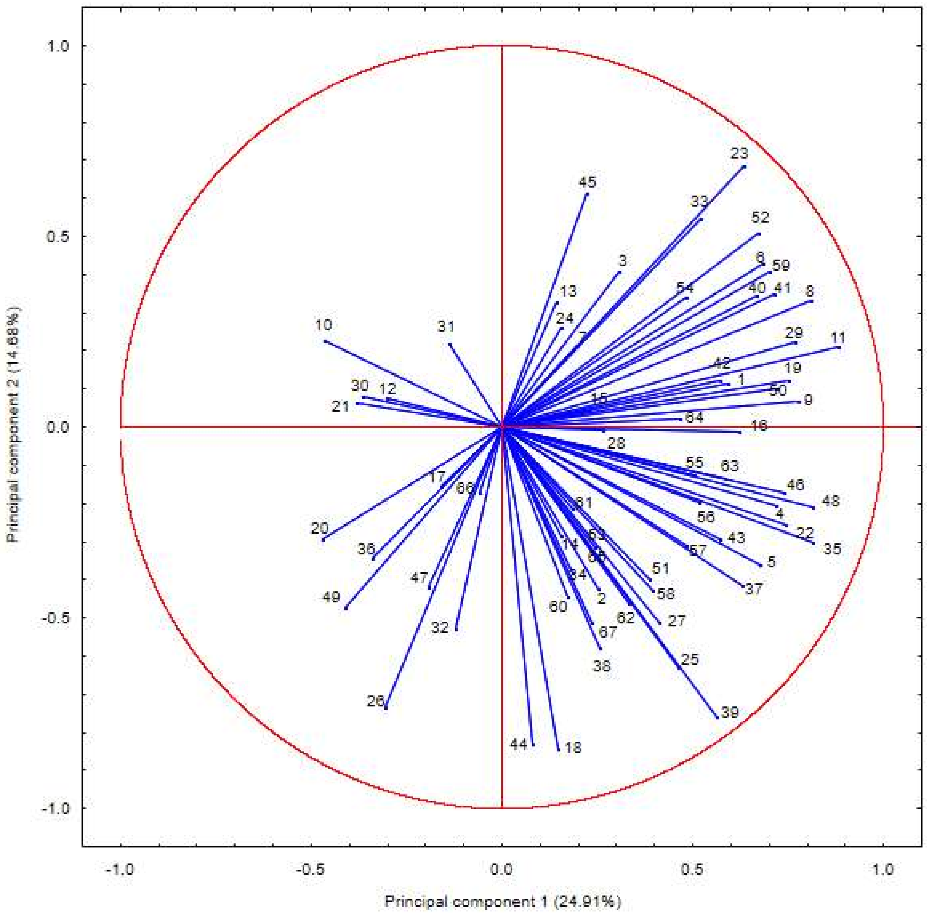

Figure S3. Projection of variables on the PCA plane defined by the first two principal components (numbers 1-67 stand for volatile compounds; see Table 1 for their names).
